# Konsensusempfehlung der ÖKG: Anatomie, Pathophysiologie, Diagnose und Therapie von Arrhythmien bei erwachsenen PatientInnen mit angeborenem Herzfehler

**DOI:** 10.1007/s00508-026-02801-0

**Published:** 2026-09-07

**Authors:** Barbara Wichert-Schmitt, Stefan Kurath-Koller, Lore Schrutka, Johannes Mair, Hermann Blessberger, Karim Saleh, Georgios Kollias, Stefan Stojkovic, Tomaz Podnar, Ante Burmas, Daniel Scherr, Christoph Schukro

**Affiliations:** 1 Klinik für Kardiologie und Internistische Intensivmedizin (Innere Medizin I), Kepler Universitätsklinikum, Med. Fakultät, Johannes Kepler Universität Linz – Campus III, Krankenhausstr. 9, Linz, Österreich; 2 Universitätsklinik für Kinder- und Jugendheilkunde, Klinische Abteilung für pädiatrische Kardiologie, Medizinische Universität Graz, Graz, Österreich; 3 Universitätsklinik für Innere Medizin II – Kardiologie, Medizinische Universität Wien, Wien, Österreich; 4 Universitätsklinik für Innere Medizin III – Kardiologie und Angiologie, Medizinische Universität Innsbruck, Innsbruck, Österreich; 5 Interne 2 – Kardiologie, Angiologie und Interne Intensivmedizin, Ordensklinikum Linz Elisabethinen, Linz, Österreich; 6 Universitätsklinik für Pädiatrie III – Kardiologie und Pulmologie, Medizinische Universität Innsbruck, Innsbruck, Österreich; 7 Universitätsklinik für Innere Medizin, Klinische Abteilung für Kardiologie, Medizinische Universität Graz, Graz, Österreich; 8 Universitätsklinik für Herz- und thorakale Aortenchirurgie, Medizinische Universität Wien, Wien, Österreich

**Keywords:** Tachyarrhythmie, Bradyarrhythmie, Antiarrhythmische Medikation, Elektrische Cardioversion, Gerätetherapie, Tachyarrhythmias, Bradyarrhythmias, Antiarrhythmic medication, Electric cardioversion, Device therapy

## Abstract

Diese Konsensusempfehlung der Arbeitsgruppe für angeborene und erworbene Herzfehler und der Arbeitsgruppe Rhythmologie der österreichischen kardiologischen Gesellschaft (ÖKG) beschreibt pathophysiologische Grundlagen, klinische Präsentation, Diagnose und Therapie von Arrhythmien bei erwachsenen PatientInnen mit angeborenem Herzfehler und beleuchtet vitienspezifische Aspekte in Bezug auf Anatomie, typische Korrektureingriffe, Arrhythmierisiko und Therapie.

## Abstract (en)

This consensus recommendation of the Working Group for Congenital and Acquired Heart Disease and the Working Group for Rhythmology of the Austrian Society of Cardiology (ÖKG) describes the pathophysiological foundations, clinical presentation, diagnosis and treatment of arrhythmias in adult patients with congenital heart defects. It also illuminates malformation-specific aspects with respect to the anatomy, typical corrective interventions, risk of arrhythmia and treatment.

## Einleitung/demografische Entwicklung

Mehr als 90 % der Neugeborenen mit angeborenem Herzfehler (AHF) erreichen heute dank herzchirurgischer, anästhesiologischer und intensivmedizinischer Fortschritte der letzten Jahrzehnte das Erwachsenenalter [1]. Folglich ist die Prävalenz von Erwachsenen mit AHF (EMAH) gestiegen und übersteigt nun bei Weitem die Anzahl von Kindern mit AHF [2]. In Deutschland leben derzeit 200.000 bis 300.000 EMAH-PatientInnen [3]. In Österreich wird die Zahl der EMAH auf etwa 25.000 geschätzt. Es wird prognostiziert, dass die Prävalenz von EMAH in den kommenden Jahren auf 1:150 ansteigen wird [2]. Die größte relative Zunahme in der Population verzeichnet dabei die Gruppe der über 50-Jährigen: Im Jahr 2030 werden ca. 11 % der EMAH älter als 60 Jahre sein [4]. Dies bedingt eine wachsende Notwendigkeit spezialisierter Versorgungsstrukturen. Rhythmusstörungen sind häufige Spätfolgen bei EMAH und mit 37 % Hauptgrund für Akutvorstellungen in Notfallambulanzen (NFA) [5]. Der plötzliche Herztod (SCD) macht etwa 20 % der Todesfälle in dieser Bevölkerungsgruppe aus [6].

## Allgemeine Grundlagen zur Ursache, Diagnose und Therapie von Arrhythmien bei EMAH

### Arrhythmiesubstrate

Die Prädisposition von EMAH zu Arrhythmien ergibt sich aufgrund angeborener Besonderheiten des Reizleitungssystems, perioperativer Verletzungen des Reizleitungssystems oder eines Koronargefäßes, postoperativer Nahtlinien, implantierter Patches, Inzisionsnarben sowie hämodynamischer und genetischer Faktoren. Um gezielte diagnostische Strategien und maßgeschneiderte Therapiekonzepte zu entwickeln, ist die Kenntnis individueller Substrate essenziell [7, 8].

### Angeborene Anomalien des Reizleitungssystems

#### Sinusknoten

Der Sinusknoten kann sowohl bei einfachen AHF – wie beim superioren Sinus-venosus-Defekt, häufiger jedoch bei komplexeren AHF – verlagert sein. Vor allem bei PatientInnen mit Heterotaxiesyndromen liegen meist ausgeprägte Sinusknotenanomalien vor. Während bei linkem Isomerismus (2 funktionell linke Vorhöfe) der Sinusknoten komplett fehlen kann, ist bei rechtem Isomerismus (2 funktionell rechte Vorhöfe) häufig ein doppelter Sinusknoten vorhanden [8–10].

#### AV-Knoten

Die Lokalisation des kompakten AV-Knotens und des His-Bündels kann abhängig vom vorliegenden kardialen Vitium deutlich variieren [10]. Eine Neigung zu kongenitalen atrioventrikulären (AV)-Blockierungen findet sich besonders bei PatientInnen mit kongenital korrigierter Transposition der großen Arterien (ccTGA) und bei PatientInnen mit linkem Isomerismus [8, 10].

#### Doppelt angelegter AV-Knoten („twin AV-node“)

Ein doppelt angelegter AV-Knoten kommt vor allem bei komplexen AHF mit AV-Diskordanz und Heterotaxiesyndromen vor. Dies kann die Entstehung von paroxysmalen supraventrikulären Tachykardien (SVT) begünstigen [8, 10].

#### Akzessorische Leitungsbahnen

Akzessorische Leitungsbahnen können bei verschiedenen AHF vorhanden sein. Die Prävalenz ist insbesondere bei PatientInnen mit Ebstein-Anomalie hoch, und häufig finden sich multiple Bahnen [7, 8]. Auch bei atrioventrikulären Septumdefekten (AVSD), Heterotaxiesyndromen, ccTGA und univentrikulären AHF treten sie vermehrt auf [8].

### Postoperative Arrhythmiesubstrate

#### Verletzungen des Reizleitungssystems

Verletzungen des Sinusknotens oder der Sinusknotenarterie im Rahmen von Operationen im Bereich des rechten Vorhofdachs können mit dem Verlust der Sinusknotenfunktion oder einer chronotropen Inkompetenz einhergehen. Dies betrifft v. a. PatientInnen mit kompletter Transposition der großen Arterien (d-TGA) nach atrialer Switch-Operation, PatientInnen nach Fontan-Operation sowie PatientInnen nach Korrekturoperationen von fehlmündenden Lungenvenen oder einem superioren Sinus-venosus-Defekt [8, 9, 11, 12]. Verletzungen des AV-Knotengewebes im Rahmen von Operationen oder Katheterinterventionen können zu AV-Blockierungen führen, wobei vor allem PatientInnen mit einer ccTGA und seltener mit AVSD gefährdet sind. Gelegentlich kommt es auch nach Patchverschluss eines perimembranösen Ventrikelseptumdefektes (VSD), operativer Sanierung von linksventrikulären Ausflusstraktstenosen oder nach Implantation von AV-Klappenprothesen zu AV-Blockierungen [8–10, 12].

#### Narben und Fremdmaterial

Chirurgische Narben nach Atriotomie und/oder Ventrikulotomie und auch das Einnähen von Patches und anderen prothetischen Materialien z. B. zum Verschluss von Vorhof- und oder Ventrikelseptumdefekten, zur Erweiterung stenotischer Strukturen (z. B. rechtsventrikuläre Ausflusstrakterweiterung bei Fallot-Tetralogie [TOF]) oder zur Umleitung des Blutflusses (Baffles – z. B. atriale Switch-Operationen) schaffen Zonen mit verlangsamter Leitung, die Makroreentrytachykardien im Vorhof (intraatriale Reentrytachykardien [IART]) und/oder Ventrikel (monomorphe ventrikuläre Tachykardien [VT]) ermöglichen [9, 12].

#### Ischämische Narben

Perioperative Verletzungen von Koronararterien, z. B. aufgrund eines abnormalen Verlaufs oder bei der Reimplantation in die Aorta können einen myokardialen Infarkt und in der Folge ischämische Narbenareale verursachen [9].

### Hämodynamische Substrate

#### Vorhofdilatation

Chronische Volumenbelastung (z. B. Shuntvitien und systemische AV-Klappeninsuffizienzen) oder Druckbelastungen (z. B. AV-Klappenstenosen oder Dysfunktion des Systemventrikels) können zur Vorhofdilatation führen und IART oder Vorhofflimmern begünstigen [13].

#### Ventrikuläre Dysfunktion und Fibrose

Chronische Druckbelastungen (z. B. Ausflusstraktstenosen, systemischer rechter Ventrikel, pulmonale Hypertonie) und Volumenbelastungen (z. B. Klappeninsuffizienzen, Shuntvitien), aber auch chronische Zyanose und myokardiale Ischämien während der Operation an der Herz-Lungen-Maschine können im Langzeitverlauf zu Hypertrophie, Dilatation und Dysfunktion der Ventrikel und diffusem fibrotischem Umbau des Myokards führen [14]. Dadurch wird das Auftreten von polymorphen VTs und Kammerflimmern begünstigt. Ein signifikant erhöhtes Risiko für ventrikuläre Arrhythmien ist besonders bei PatientInnen mit Dysfunktion des subaortal gelegenen Ventrikels (z. B. systemischer rechter Ventrikel bei d‑TGA nach Vorhofumkehroperation), Dysfunktion des subpulmonal gelegenen rechten Ventrikels (z. B. TOF), nach Fontan-Operation und PatientInnen mit Eisenmenger-Syndrom vorhanden [8, 15].

#### Dyssynchronie

Auch eine dyssynchrone Ventrikelaktivierung kann die Entstehung einer ventrikulären Dysfunktion fördern und konsekutiv das Risiko für VT erhöhen. Bei AHF sind Schenkelblockbilder meist Folge einer Verletzung des Reizleitungssystem während der Korrekturoperation. Ein Rechtsschenkelblock ist häufiger als ein Linksschenkelblock, wie z. B. nach VSD-Patchverschluss oder Korrekturoperation einer TOF. [9, 16].

## Präsentation und Evaluierung von EMAH mit Arrhythmien in der Notfallambulanz

Arrhythmien sind die häufigste Ursache für Akutvorstellungen von EMAH in der NFA [17]. Die PatientInnen geben häufig unspezifische Symptome wie Dyspnoe, Palpitationen oder thorakale Schmerzen an [18]. Arrhythmien sind auch die häufigste Ursache für Synkopen und Präsynkopen bei EMAH [17]. Eine *genaue Anamnese* in Bezug auf die kardiale Vorgeschichte ist essentiell. Diese soll frühere Operationen, Implantate (z. B. Shunts, Patches, Conduits), vorbestehende EKG-Veränderungen wie Schenkelblockbilder, und vorbekannte Rhythmusstörungen umfassen. Bereits laufende Therapien wie antiarrhythmische Medikation, ein implantierter Schrittmacher (PSM) oder implantierbarer Kardioverter-Defibrillator (ICD) müssen erfragt werden. Bei der klinischen Untersuchung sollte auf Zeichen der hämodynamischen Instabilität und kardialen Dekompensation geachtet werden, um eine akute Therapienotwendigkeit zu erkennen [8]. Umgehend muss ein 12-Kanal-Elektrokardiogramm (EKG) aufgezeichnet werden und – falls vorhanden – der PSM, ICD oder Loop-Rekorder abgefragt werden [17].

### Cave

*Jede Arrhythmie – Brady- und Tachyarrhythmie – kann bei EMAH-PatientInnen mit komplexen Vitien zu einer raschen klinischen Verschlechterung und/oder hämodynamischen Instabilität führen.*

*Eine frühzeitige Kontaktaufnahme mit dem behandelnden EMAH-Zentrum sollte erfolgen um weitere notwendige Behandlungsschritte zu besprechen und, falls erforderlich, eine Verlegung zu organisieren.*

### Tachyarrhythmien

Bei EMAH-PatientInnen kann die Unterscheidung zwischen SVT und VT aufgrund eines vorbestehenden breiten QRS-Komplexes oder einer Tachykardie-assoziierten aberranten Leitung oft schwierig sein, weswegen ein Vergleich mit einem Vor-EKG hilfreich ist. Idealerweise führen PatientInnen eine elektronische Kopie ihres Ruhe-12-Kanal-EKGs mit sich [17]. Im Zweifelsfall sollte jede Tachykardie mit breitem Kammerkomplex > 120 ms wie eine VT behandelt werden. Zu beachten ist, dass – abhängig vom zugrunde liegenden kardialen Vitium – auch eine persistierende atriale Tachykardie (AT) zu einer kardialen Dekompensation, hämodynamischen Instabilität oder SCD führen kann. Bei hämodynamischer Instabilität muss eine sofortige elektrische Kardioversion erfolgen. In stabilen Situationen kann eine medikamentöse Therapie erwogen werden, allerdings ist die Datenlage für antiarrhythmische Medikation bei EMAH schwach. Die höchste Sicherheit für die PatientInnen gewährleistet demnach die Kardioversion [17]. Eine Dokumentation des initialen Herzrhythmus mit einem 12-Kanal-EKG vor Gabe von Antiarrhythmika und vor anderen medizinischen Interventionen ist für die weitere elektrophysiologische Beurteilung, einschließlich der Eignung für eine Ablationstherapie, von größter Bedeutung [8, 17]. Schilddrüsen‑, Nieren- und Leberfunktion, Blutbild und die Gerinnungsparameter sollten bestimmt werden [8, 18]. Eine weitere Evaluierung durch KardiologInnen mit Erfahrung in der Therapie von PatientInnen mit angeborenen Herzfehlern sollte erfolgen, um anatomische Probleme, die Trigger für Arrhythmien darstellen und saniert werden können, zu erkennen. Elektrolytstörungen müssen ausgeglichen und Infekte behandelt werden [8, 18].

### Therapie von Tachyarrhythmien

#### 1. Vagale Manöver

Eine Karotisdruckmassage oder ein modifiziertes Valsalva-Manöver kann bei regelmäßigen SVT versucht werden. AV-Knoten-abhängige Reentrytachykardien können hierdurch terminieren. Im Falle von IARTs kann durch Verlangsamung der Ventrikelfrequenz die atriale Aktivität demaskiert werden [19].

#### 2. Elektrische Kardioversion

Die elektrische Kardioversion (eCV) ist eine sichere und effektive Methode zur Terminierung von SVT und VT bei EMAH. Bei PatientInnen mit komplexen AHF besteht eine erhöhte Neigung zur Thrombusformation, daher sollten – falls nicht schon zumindest ≥ 4 Wochen eine orale Antikoagulation eingenommen wurde – intrakardiale Thromben mittels transösophagealer Echokardiographie vor der Durchführung der eCV ausgeschlossen werden, es sei denn, eine akute Kardioversion ist aufgrund einer hämodynamischen Instabilität erforderlich [7–9]. Weiters sollte man vorbereitet sein, dass im Anschluss an die eCV eine chronotrope und/oder inotrope Therapie erforderlich sein kann, da gleichzeitig eine Neigung zu Bradykardien oder eine Ventrikeldysfunktion vorliegen können ([8, 9]; s. Tab. 1, Checkliste eCV).

**Tab. 1:** Checkliste für elektrische Kardioversion

**PatientInnenevaluation:**	
1. 12-Kanal-EKG:	– Dokumentation der Arrhythmie – Vergleich mit Vor-EKG - SVT oder VT?
2. Hämodynamische Instabilität oder kardiale Dekompensation?	– Klinik, Blutdruck, Sauerstoffsättigung
3. Arztbrief aus EMAH-Ambulanz vorhanden?	– Kenntnis des AHF und vorausgegangener Korrektureingriffe – Antiarrhythmische Medikation? – Adäquate Antikoagulation? – Device-Therapie (PSM, ICD, Loop-Recorder) vorhanden?
4. Echokardiographie:	– biventrikulärer oder univentrikulärer AHF? – Ventrikelfunktion (subaortaler und subpulmonaler Ventrikel)? – Herzklappenfunktion? – Vorhofdilatation?
5. Transösophageale Echokardiographie:	– zum Ausschluss intrakardialer Thromben wenn erforderlich – nicht notwendig bei hämodynamischer Instabilität
**Monitoring:**	
1. EKG	
2. Sauerstoffsättigung	– Vergleich mit Ausgangswert aus dem EMAH Arztbrief – Luftfilter auf iv Zugänge bei Hypoxämie (Rechts-Linksshunt)
3. Blutdruck:	– auf beiden Armen messen (Cave möglicher Verschluss der Arteria subclavia z. B. nach Operation einer Aortenisthmusstenose)
**Vorbereitung auf mögliche Komplikationen: Risiko für Bradykardie und hämodynamische Instabilität nach der eCV?**	
1. Möglichkeit zum raschen Beginn einer chronotropen und/oder inotropen Medikation (29)	– Atropin 0,5 mg iv bis max 3 mg – Isoprenalin 5mcg/min iv – Therapie des kardiogenes Schocks gemäß ESC Leitlinie (35)
2. Möglichkeit zur Initiierung einer passageren Schrittmachertherapie	Cave: kein oder schwieriger venöser Zugang zum Herz (Fontan-Zirkulation, Glenn-Shunt, dTGA nach atrialer Switch Operation) – Transkutane Stimulationsmöglichkeit vorbereiten
3. Reanimationsbereitschaft	
**Sedierung/Anästhesie:**	
Durchführung idealerweise durch ein erfahrenes Anästhesieteam bei komplexen AHF	
**Elektrische Kardioversion- Technik:**	
1. Anbringen der Elektroden:	Cave: die Lage des Herzens im Thorax muss berücksichtigt werden: Situs inversus, Dextrokardie oder Mesokardia? Antero-posteriore Elektrodenplatzierung bei komplexen AHF und dilatierten Vorhöfen
2. Biphasischer Defibrillator:	mit maximalem Energieniveau, um rezidivierende Schockabgaben zu vermeiden
3. Durchführung:	Synchronisierte Schockabgabe auf die R-Zacke (außer Defibrillation bei Kammerflimmern)
4. 12-Kanal-EKG	Dokumentation des Rhythmus nach Kardioversion
**Nachsorge:**	
**Überwachung:**	– Bradykardie? – Arrhythmierezidiv? – Hämodynamische und respiratorische Stabilität?
**Langzeit-Management:**	
Kontaktaufnahme mit dem behandelnden EMAH-Zentrum	– **Überstellung notwendig oder Vereinbarung einer ambulanten Kontrolle?** – Korrigierbare Ursache? : Infektion, Elektrolytverschiebungen, Schilddrüsenfunktionsstörungen – Anatomisch/strukturelle Komplikation? – Medikamentöse Therapie zur Rhythmuskontrolle? – Elektrophysiologische Untersuchung und Ablation? – Indikation zur Gerätetherapie? (PSM,ICD)

*EKG* Elektrokardiogramm; *SVT* Supraventrikuläre Tachykardie; *VT* Ventrikuläre Tachykardie; *EMAH* Erwachsene mit angeborenem Herzfehler; *AHF* Angeborener Herzfehler; *PSM* Implantierter Schrittmacher; *ICD* Implantierter Cardioverter Defibrillator; *iv* intravenös; *mg* Milligramm; *mcg/min* Mikrogramm pro Minute; *dTGA* Komplette Transposition der großen Arterien

#### 3. Medikamentöse Therapie

Aufgrund der Heterogenität der PatientInnen mit AHF liegen keine Daten aus randomisierten Studien hinsichtlich medikamentöser Therapie von Arrhythmien bei EMAH vor. Die Tab. 2 listet Medikamente zur Akuttherapie von SVT, Tab. 3 Medikamente zur Akuttherapie von VT auf. Die Erfolgsrate einer antiarrhythmischen Medikation als langfristige Therapie ist eingeschränkt und die Neigung zu kardialen und extrakardialen Nebenwirkungen erhöht [7, 8]. Vor Verabreichung einer antiarrhythmischen Medikation sollte eine Echokardiographie durchgeführt werden.

#### a) Supraventrikuläre Tachyarrhythmien

##### i) Antiarrhythmische Medikamente zur Akuttherapie supraventrikulärer Tachyarrhythmien

In der Tab. 2 sind Medikamente zur Akuttherapie von SVT aufgelistet.

##### ii) Antiarrhythmika im Langzeitmanagement supraventrikulärer Tachyarrhythmien

Der Erhalt eines Sinusrhythmus sollte bei EMAH unbedingt angestrebt und [18] eine Frequenzkontrolle als Langzeittherapieziel nur bei Versagen anderer Therapieoptionen akzeptiert werden [8]. Bei der Wahl der antiarrhythmischen Therapie müssen folgende Faktoren mitberücksichtigt werden: gleichzeitig vorliegende Sinusknoten- oder AV-Knotendysfunktion, systemische oder subpulmonale Ventrikeldysfunktion, andere medikamentöse und Gerätetherapien, mögliche Schwangerschaft und erworbene Komorbiditäten (z. B. Niereninsuffizienz) [9]. Generell ist eine Ablationsstrategie im Falle von zugänglichen Arrhythmiesubstraten einer medikamentösen Langzeittherapie vorzuziehen [18].

**Tab. 2:** Antiarrhythmika zur Akuttherapie supraventrikulärer Tachyarrhythmien

Adenosin	6–18 mg i.v.-Bolus über großlumigen i.v.-Zugang in der Cubita, mit NaCl-Bolus nachspülen wird zur Terminierung von AV-Knoten-abhängigen SVTs empfohlen [8]; kann auch bei unklarer Tachyarrhythmie Flatterwellen bei IART demaskieren. NW: Bronchospasmus, Vasodilatation, Antidot: Theophyllin
i.v.-Betablocker	Zur Frequenzkontrolle vor einer möglichen spontanen Kardioversion bei hämodynamisch stabilen PatientInnen
	Eine langsame Infusion ist einer i.v.-Bolusgabe vorzuziehen
	Können bei PatientInnen mit eingeschränkter Ventrikelfunktion aufgrund der negativ inotropen Wirkung zur hämodynamischen Verschlechterung führen [8]
	Z. B. Metoprolol 2,5–5 mg langsam i.v., Wiederholung nach 5–10 min möglich. (Gesamtdosis maximal 10–15 mg)
Non-Dihydropyridin-Kalziumkanalantagonisten	Kontraindiziert unter anderem bei einer EF < 40 %
Amiodaron	150–300 mg i.v., in 5 %iger Glukose als Kurzinfusion; kann bei hämodynamisch stabilen PatientInnen mit strukturellen Auffälligkeiten und ventrikulärer Dysfunktion eingesetzt werden [17]. In der Akuttherapie zur medikamentösen Kardioversion und auch als Rezidivprophylaxe. Als Ultima Ratio auch zur Frequenzkontrolle bei tachykardem Vorhofflimmern oder atrialen Makro-Reentrytachykardien
Ibutilide	1 mg initial über 10 min., es kann danach bei fehlender Wirkung eine weitere Dosis von 1mg über 10 min. verabreicht werden unter Monitoring zur Kardioversion von Vorhofflattern und -flimmern, Beenden der Infusion bei Konversion in den Sinusrhythmus, QT-Verlängerung oder ventrikulären Arrhythmien [19, 21]
Vernakalant	3 mg/kg KG über 10 min., bei fehlender Wirkung nach 15 min. nach Erstgabe kann eine 2. Dosis mit 2 mg/kg KG über 10 min. verabreicht werden zur Kardioversion von Vorhofflimmern innerhalb der ersten 10 Tage (gegebenenfalls postoperativ innerhalb der ersten 3 Tage); bei komplexen Vitien nicht untersucht [21]

*i.v.* intravenös, *NaCl* Natriumchlorid, *SVT* supraventrikuläre Tachykardie, *IART* intraatriale Reentrytachykardie, *NW* Nebenwirkungen, z. B. zum Beispiel, *mg* Milligramm, *EF* Ejektionsfraktion, *kg* Kilogramm

Details zu Verabreichung, Nebenwirkungen und Kontraindikation sind in der jeweiligen Medikamenten-Fachinformation nachzulesen

**Tab. 3:** Antiarrhythmika zur Akuttherapie ventrikulärer Tachyarrhythmien

*Eine instabile und auch stabile ventrikuläre Tachykardie sollte, wenn möglich, mittels eCV terminiert werden.***Eine ausreichende Datenlage zum Einsatz antiarrhythmischer Medikamente bei EMAH liegt nicht vor**	
*Amiodaron*	Kann bei stabiler VT zur elektrischen Kardioversion eingesetzt werden, wenn eine elektrische Kardioversion nicht möglich ist [8, 21]
*Procainamid* – in Österreich nicht verfügbar	Kann bei stabiler VT zur elektrischen Kardioversion eingesetzt werden, wenn eine elektrische Kardioversion nicht möglich ist [8, 21]
*Ajmalin*	Sehr viele Kontraindikationen [21]
*Lidocain*	Reservemedikament, als Akuttherapie bei Schock-refraktärem VF oder pulsloser ventrikulärer Tachykardie [21]

*eCV* elektrische Kardioversion, *EMAH* Erwachsene mit angeborenem Herzfehler, *VT* ventrikuläre Tachykardie, *VF* Kammerflimmern

Details zu Verabreichung, Nebenwirkungen und Kontraindikation sind in der jeweiligen Medikamenten-Fachinformation nachzulesen

##### Rhythmuskontrolle

Betablocker sind Medikamente der ersten Wahl und werden bei PatientInnen mit d‑TGA nach atrialer Switchoperation mit SVT auch zur Prävention ventrikulärer Arrhythmien empfohlen [20]. Amiodaron ist ein wirksames Antiarrhythmikum und kann auch bei ventrikulärer Dysfunktion oder komplexen AHF eingesetzt werden, die Anwendung ist jedoch limitiert durch die Langzeittoxizität. Die Amiodaron-induzierte Thyreotoxikose ist vor allem bei PatientInnen mit zyanotischen AHF, niedrigem Body-Mass-Index < 21 und Fontan-Zirkulation häufig [8, 9]. Sotalol wird bei EMAH aufgrund erhöhter Proarrhythmogenität primär nicht empfohlen und kann nur verabreicht werden, wenn keine schwere ventrikuläre Dysfunktion vorliegt. Orale Klasse-I-Antiarrhythmika (Flecainid/Propafenon) sollen bei EMAH mit eingeschränkter systemischer oder subpulmonaler Ventrikelfunktion, Sinusknoten- oder AV-Knotendysfunktion, breitem QRS-Komplex und/oder koronarer Herzkrankheit nicht eingesetzt werden. Zu beachten ist auch, dass diese Medikamente aufgrund einer Leitungsverzögerung auf Vorhof- und Ventrikelebene einerseits zu Bradykardien, andererseits bei einer Verlangsamung der Zykluslänge bei IARTs zu einer 1:1-Überleitung über den AV-Knoten und damit zu einer Akzeleration der Ventrikelfrequenz führen können [8, 9].

##### Frequenzkontrolle

Zur Frequenzkontrolle werden vor allem Betablocker eingesetzt. Eine Alternative stellen Kalziumantagonisten (Verapamil/Diltiazem) dar, sollen aber nur bei EMAH ohne Präexzitation und ohne Ventrikeldysfunktion verwendet werden. Digoxin wird nicht mehr als Erstlinien- und nicht als Monotherapie empfohlen [8, 9].

#### b) Ventrikuläre Tachyarrhythmien

Eine instabile und auch stabile ventrikuläre Tachykardie sollte mittels eCV terminiert werden, wenn möglich nach vorhergehender Aufzeichnung eines 12-Kanal-EKGs. Nach einer instabilen ventrikulären Tachykardie, nicht-idiopathischen VT oder nach überlebtem SCD besteht eine Klasse-I-Indikation zur Implantation eines ICD, eine alleinige medikamentöse antiarrhythmische Therapie ist nicht empfohlen [8].

##### i) Antiarrhythmika zur Akuttherapie ventrikulärer Tachyarrhythmien

In der Tab. 3 sind Medikamente zur Akuttherapie von VT aufgelistet [21].

##### ii) Antiarrhythmika im Langzeitmanagement ventrikulärer Tachyarrhythmien

Betablocker werden zur Behandlung von nicht anhaltenden ventrikulären Arrhythmien zur symptomatischen Therapie und zur Reduktion des Risikos für eine Verschlechterung der ventrikulären Funktion empfohlen [8]. Antiarrhythmika (Amiodaron und Betablocker) können auch zur Reduktion von VT-Episoden bei PatientInnen mit ICD eingesetzt werden [8].

*Bei EMAH PatientInnen stellt die antiarrhythmische Therapie in vielen Fällen eine zeitlich begrenzte Überbrückung dar; bei zugänglichem Substrat ist die Ablation der bevorzugte definitive Therapieansatz.*

### Orale Antikoagulation

Das Risiko für thromboembolische Komplikationen ist bei EMAH aufgrund von häufigen atrialen Arrhythmien, residuellen Klappenvitien, Verwendung von prothetischem Material im Rahmen der Korrektur, persistierenden intrakardialen Shunts, Zyanose oder ventrikulärer Dysfunktion signifikant höher im Vergleich zur Allgemeinbevölkerung [18, 22, 23]. Eine orale Antikoagulation (OAK) ist indiziert bei Auftreten von Vorhofflimmern, Vorhofflattern und hochfrequenten atrialen Tachykardien nach Abwägung des Thromboembolie- und des Blutungsrisikos [8]. Die Anwendung des CHA_2_DS_2_-VA-Score zur Indikationsstellung einer OAK-Therapie wird allerdings nur bei EMAH mit „leichten“ angeborenen Herzfehlern (s. Tab. 4) empfohlen, bei PatientInnen mit „mittelschweren“ und „schweren“ AHF wird zur Einleitung einer OAK geraten [7, 8]. Durch die Einführung der „Nicht-Vitamin-K-Antagonisten“ (neue bzw. direkte orale Antikoagulanzien [NOAKs]) als neue Standardtherapie von thromboembolischen Erkrankungen und Vorhofflimmern hat sich die OAK in den letzten 15 Jahren wesentlich geändert. Jedoch wurden bei allen großen klinischen Studien, die zur Zulassung dieser Medikamente führten, EMAH-PatientInnen nicht eingeschlossen. Es gibt daher nur wenige Daten zum Vergleich von Vitamin-K-Antagonisten und NOAKs in diesem PatientInnenkollektiv. Deshalb werden in den Richtlinien immer noch Vitamin-K-Antagonisten als Standardtherapie angeführt [18]. Dennoch werden laut Registerdaten NOAKs auch bei EMAH-PatientInnen zunehmend häufiger eingesetzt [22, 23]. Vitamin-K-Antagonisten sind nach wie vor die Therapie 1. Wahl bei mechanischen Herzklappenprothesen, durch mittel- oder höhergradige Mitralklappenstenosen bedingtem Vorhofflimmern oder bei speziellen Gerinnungsstörungen (z. B. Antiphospholipidsyndrom) [24, 25]. Zusätzlich fehlen meist noch ausreichende Sicherheitsdaten zum Einsatz der NOAKs bei PatientInnen mit schwerer Nieren- oder Leberinsuffizienz [24, 25].

**Tab. 4:** Einteilung angeborener Herzfehler (gemäß ESC Guidelines)

***Einfacher angeborener Herzfehler***
Isoliertes kongenitales Vitium der Aortenklappe, bikuspide Aortenklappe
Isoliertes kongenitales Vitium der Mitralklappe (außer „parachute mitral valve“ und Cleft der Mitralklappen)
Geringe isolierte Pulmonalstenose
Isolierter kleiner Atriumseptumdefekt (ASD), Ventrikelseptumdefekt (VSD) oder persistierender Ductus arteriosus Botalli (PDA)
Korrigierter ASD vom Secundum-Typ, Sinus-venosus-Defekt, VSD oder PDA ohne Residuen oder Spätkomplikationen wie ventrikuläre Dilatation, Dysfunktion oder pulmonale Hypertension
***Moderater angeborener Herzfehler (korrigiert oder nicht korrigiert)***
Partielle oder komplette Lungenvenenfehlmündung
Koronararterienanomalien: Abgang einer Koronararterie von der Pulmonalarterie oder vom gegenüberliegenden Sinus
Aortenstenose (subvalvulär, supravalvulär)
Atrioventrikulärer Septumdefekt (komplett oder partiell) inklusive ASD vom Primum-Typ
ASD vom Secundum-Typ (nicht korrigiert, moderat bis groß)
Aortenisthmusstenose
„Double chambered right ventricle“
Ebstein-Anomalie
Marfan-Syndrom und andere hereditäre thorakale Aortenerkrankungen, Turner-Syndrom
PDA (nicht korrigiert, moderat bis groß)
Periphere Pulmonalstenose
Pulmonalstenose (infundibulär, valvulär, supravalvulär; mittelschwer bis schwer)
Sinus-venosus-Defekt (nicht korrigiert)
Sinus-Valsalva-Aneurysma/Fistel
Fallot-Tetralogie (korrigiert)
Komplette Transposition der großen Gefäße (nach arterieller Switch-Operation)
VSD mit assoziierten Fehlbildungen und/oder unkorrigiert mit moderatem bis großem Shunt (ohne pulmonalarterielle Hypertonie)
***Komplexer angeborener Herzfehler: (korrigiert oder nicht korrigiert, sofern nicht anders spezifiziert)***
Jeglicher AHF mit pulmonalvaskulärer Erkrankung inklusive Eisenmenger-Syndrom
Jeglicher zyanotischer AHF (unkorrigiert oder palliiert)
„Double outlet ventricle“ (meist „double outlet right ventricle“)
Fontan-Zirkulation
Unterbrochener Aortenbogen
Pulmonalatresie (alle Formen)
Transposition der großen Arterien (außer nach arterieller Switch-Operation)
Univentrikuläre Herzen
Truncus arteriosus communis
Andere komplexe Fehlbildungen einer atrioventrikulären und ventrikuloarteriellen Verbindung (z. B. Criss-cross-Herz, Heterotaxiesyndrome, invertierte Ventrikel)

*ESC* European Society of Cardiology, *ASD* Atriumseptumdefekt, *VSD* Ventrikelseptumdefekt, *PDA* persistierender Ductus arteriosus Botalli, *AHF* angeborener Herzfehler

Es gibt zwar keine randomisierten kontrollierten Studien zum Einsatz der NOAKs im Vergleich zu Vitamin-K-Antagonisten bei EMAH-PatientInnen, jedoch lassen sich die vorliegenden Daten basierend auf einer Metaanalyse wie folgt zusammenfassen [26]:

1. Vitamin-K-Antagonisten und NOAKs waren vergleichbar effektiv zur Verhinderung von thromboembolischen Komplikationen, wobei bei Fontan-PatientInnen in einer Studie thromboembolische Komplikationen unter Vitamin-K-Antagonisten seltener auftraten.
2. Es gab keinen signifikanten Unterschied bei Blutungskomplikationen, wobei intrakranielle Blutungen unter NOAKs tendenziell seltener auftraten.

### Elektrophysiologische Abklärung und Ablation

Eine Katheterablation ist bei rezidivierenden Tachyarrhythmien als First-line-Therapie empfohlen und einer langfristigen medikamentösen Therapie vorzuziehen, auch wenn die Rezidivrate aufgrund des häufig komplexen Substrates erhöht ist [8, 18]. Eine sorgfältige Planung der elektrophysiologischen Untersuchung ist essenziell, hierfür ist eine Dokumentation der Tachykardie mittels 12-Kanal-EKG sehr hilfreich. Wenn mehrere verschiedene Rhythmusstörungen vorliegen, kann dies mittels Langzeit-EKG und auch Wearables detektiert werden [27]. Eine Ablation bei laufender Tachykardie ermöglicht, den Mechanismus zu identifizieren und gezielt zu behandeln. Insofern kann im Falle einer hämodynamisch gut tolerierten SVT Kontakt mit dem behandelnden EMAH-Zentrum/Rhythmologie aufgenommen werden, um die Möglichkeit einer kurzfristigen Ablation zu evaluieren. Eine genaue Kenntnis der Anatomie des Herzfehlers, der möglichen assoziierten Anomalien des Reizleitungssystems sowie der bereits durchgeführten Operationen und Interventionen ist erforderlich [8, 9]. Frühere Befunde, Operations- und Interventionsberichte sind hierfür einzuholen. Mittels vorhergehender 3‑dimensionaler (3D) Bildgebung (Magnetresonanztomographie [MRT] oder Computertomographie [CT]) können die räumliche Beziehung verschiedener kardialer Strukturen zueinander und deren Dimensionen dargestellt werden [27]. Auch müssen (Gefäß‑)Zugänge zu den jeweiligen Herzkammern vor dem Eingriff evaluiert und geplant werden. Hierbei kann es notwendig sein, intrakardiale Strukturen – wie z. B. Baffles – zu punktieren, um zum Arrhythmiesubstrat zu gelangen [27, 28]. Die Verwendung von 3D-Mapping-Systemen ist unerlässlich, und Maßnahmen zur Reduktion der Durchleuchtungszeit müssen unbedingt getroffen werden [8]. Als institutionelle Voraussetzungen für die Behandlung von Arrhythmien bei PatientInnen mit AHF wird zusätzlich zu entsprechend spezialisierten ElektrophysiologInnen das Vorhandensein einer Herzchirurgie, eines Herzkatheterlabors sowie einer Intensivmedizin mit Personal mit Erfahrung in der Behandlung von AHF gefordert [8]. In speziellen anatomischen Situationen (Fontan mit extrakardialem Conduit, d‑TGA nach atrialer Switchoperation) ist es einfacher, das Arrhythmiesubstrat mittels magnetischem Navigationssystem zu erreichen [27, 28], allerdings ist diese technische Möglichkeit nur an vereinzelten Zentren vorhanden.

### Bradyarrhythmien

#### a) Akuttherapie von symptomatischen Bradyarrhythmien

Zur Akuttherapie von Bradyarrhythmien kann Atropin oder auch eine Isoprenalin-Infusion verabreicht werden, Bradykardie-fördernde Medikamente sollen abgesetzt werden [17, 29]. Im Falle einer symptomatischen Bradykardie, die eine akute Anlage eines passageren Schrittmachers erfordert, sollten technische Schwierigkeiten bei der Implantation eingeplant werden. Daneben sollte die Möglichkeit zur transkutanen Stimulation oder – wenn vorhanden – einer Sondenlegung retrograd über die Aorta in den Ventrikel bereitgehalten werden [17] (s. Kapitel Schrittmachertherapie). Bei PatientInnen mit komplexen AHF kann es sinnvoll sein – nach Rücksprache mit dem behandelnden EMAH-Zentrum – einen Transfer zur weiteren Therapie zu organisieren [17].

#### b) Vorhofbradykardien

Eine Sinusbradykardie, ein junktionaler Ersatzrhythmus oder ein inadäquater Anstieg der Vorhoffrequenz (chronotrope Inkompetenz) bei körperlicher Aktivität finden sich häufig als Ursache einer verminderten Leistungsfähigkeit. Auch vermeintlich asymptomatische Vorhofbradykardien können zu einem atrialen Remodeling führen und aufgrund der verlängerten Diastolendauer das Auftreten atrialer Arrhythmien begünstigen. Der Verlust der AV-Synchronität kann längerfristig die Entwicklung einer Herzinsuffizienz oder AV-Klappeninsuffizienz verursachen [9, 18, 20]. Eine Schrittmachertherapie wird bei isolierter Sinusknotendysfunktion empfohlen: im Falle einer symptomatischen Bradykardie, Verlust der AV-Synchronität, Belastungsintoleranz bei chronotroper Inkompetenz oder Bradykardie-assoziierter hämodynamischer Beeinträchtigung [8, 9, 18]. Bei Brady-Tachykardie-Syndromen kann eine vorsichtige Erhöhung der Vorhoffrequenz mittels atrialer Stimulation eine Reduzierung der Tachykardieepisoden bewirken und Teil eines multimodalen Therapieansatzes sein [8, 9, 18]. Bei PatientInnen mit komplexen AHF soll eine permanente Schrittmacherimplantation auch bei einer Sinusbradykardie mit einer Ruhefrequenz < 40/min, symptomatischen Pausen > 3 s oder asymptomatischen Pausen > 6 s erwogen werden. Die Indikationsstellung muss jedoch nach sorgfältiger Nutzen-Risiko-Abwägung im Rahmen einer multidisziplinären Teamdiskussion an einem erfahrenen EMAH Zentrum erfolgen. [8, 18].

#### c) AV-Blockierungen

Symptomatische AV-Blockierungen stellen – unabhängig von ihrem Grad – eine Schrittmacherindikation dar [8, 9, 18, 30]. Für asymptomatische PatientInnen mit einem „höhergradigen AV-Block“ (d. h. mindestens 2 aufeinanderfolgende nicht übergeleitete P‑Wellen aufgrund eines AV-Blocks Grad II Typ Mobitz oder Grad III) wird eine PSM-Therapie empfohlen, wenn entweder eine Ventrikeldysfunktion, ein reduziertes Herzzeitvolumen, ein Ersatzrhythmus mit breitem Kammerkomplex, komplexe ektope ventrikuläre Schläge oder ein verlängertes QT-Intervall bestehen [8, 30]. Postoperative höhergradige AV-Blockierungen führen zu einem erhöhten Risiko für SCD. Eine Schrittmacherimplantation wird unabhängig von klinischen Symptomen empfohlen, wenn innerhalb von mindestens 5 Tagen nach einer kardialen Operation keine Erholung des Reizleitungssystems eintritt [8, 9, 18, 30], ebenso bei PatientInnen mit perioperativer transienter kompletter AV-Blockierung und persistierendem bifaszikulärem Block [8, 9, 30]. In Einzelfällen kann bei EMAH mit Synkopen und hoher Vortestwahrscheinlichkeit für Erkrankung des Reizleitungssystems eine elektrophysiologische Untersuchung und Messung der HV-Zeit erwogen werden [31].

### Schrittmachertherapie

Bei der Planung der Implantation eines PSM- und/oder ICD-Systems bei EMAH sind verschiedene Aspekte zu beachten, welche zuvor im multidisziplinären Team aus den Fachbereichen EMAH-Kardiologie, invasive Elektrophysiologie, Device-Therapie und Herzchirurgie besprochen werden sollten [9, 30].

#### Kardiale Anatomie

Eine detaillierte Kenntnis der vorliegenden kardialen Anatomie und zuvor durchgeführten Operationen und/oder interventionellen Eingriffen ist unbedingt erforderlich. Ein Situs inversus, die Position des Herzens im Thorax (z. B. Dextrokardie), die Anatomie der Vorhöfe (z. B. Heterotaxiesyndrome), ein Malalignement von Vorhöfen und Ventrikel mit Verlagerungen des Reizleitungssystems, die AV-Klappenmorphologie (z. B. apikale Verlagerung des Trikuspidalklappenanulus bei Morbus Ebstein), die Ventrikelmorphologie (u. a. anatomisch rechter oder linker Ventrikel, Hypoplasie, Hypertrophie, Fibrose) und intrakardiale Shunts müssen bekannt sein. Aus der Durchsicht von Operations- und Katheterberichten kann in Erfahrung gebracht werden, ob und wo sich Patches zum Verschluss von ASD oder VSD, interventionell eingebrachte Okkluder, Baffles (z. B. bei Z. n. atrialer Switch-Operation), Klappenprothesen, Conduits oder auch frühere Schrittmachersonden befinden [9, 30].

#### Venöser Zugang

Ein unkomplizierter venöser Zugang zum Herz kann bei EMAH nicht vorausgesetzt werden. Venöse Kanülierungen im Rahmen von Operationen und Herzkathetereingriffen im jungen Alter können zu chronischen Venenverschlüssen führen, ebenso wie bereits früher eingebrachte PSM- oder ICD-Sonden. Anatomische Varianten wie eine persistierende linke obere Hohlvene, fehlende V. anonyma, ein Coronarsinusdefekt oder Baffle-Stenosen bei Z. n. atrialer Switch-Operation müssen im Vorhinein bedacht werden. Eine Phlebographie oder bei spezieller Fragestellung erweiterte Bildgebung mit MRT oder CT werden zur Abklärung empfohlen [9, 30]. Bei PatientInnen nach einer Fontan-Operation (heutzutage meist mit einem extrakardialen Conduit) muss damit gerechnet werden, dass kein venöser Zugang in das Herz vorhanden ist, da die V. cava superior (SVC) und V. cava inferior (IVC) direkt mit den Pulmonalarterien anastomosiert wurden [9, 30].

#### Bestimmung des adäquaten Schrittmachersystems

Bei EMAH können gleichzeitig sowohl eine Sinus- als auch AV-Knotendysfunktion sowie zusätzlich eine Dyssynchronie und/oder Herzinsuffizienz bestehen. Eine genaue rhythmologische und strukturell/funktionelle kardiale Evaluierung muss zur Bestimmung des adäquaten Schrittmachersystems (Ein- vs. Zwei-Kammer-Schrittmacher, kardiale Resynchronisationstherapie [CRT], ggf. zusätzliche ICD-Funktion) erfolgen [8, 9, 30].

#### Pacing Site

Eine Stimulation des Apex oder der freien Wand des subpulmonalen Ventrikels kann aufgrund der dadurch verursachten Dyssynchronie zu einer Funktionsverschlechterung des Systemventrikels führen. Wurde ein VSD mittels Patch verschlossen, kann eine septale Positionierung der Ventrikelsonde erschwert sein. Bei Anzeichen einer Funktionsverschlechterung des Systemventrikels unter univentrikulärer Stimulation sollte frühzeitig auf eine kardiale Resynchronisationstherapie (CRT) aufgerüstet werden. Für Personen mit anatomisch rechtem Systemventrikel wird primär zur Implantation eines biventrikulären Schrittmachersystems geraten. Hierzu muss die Möglichkeit der Sondierung des Koronarsinus im Vorhinein abgeklärt werden [9, 30]. Bei bis zu einem Drittel der EMAH müssen epikardiale Schrittmachersysteme implantiert werden. Gründe hierfür sind neben fehlender venöser Zugänge offene intrakardiale Shunts (Thromboembolierisiko), Klappenprothesen, schwere AV-Klappeninsuffizienzen oder spezifische Überlegungen hinsichtlich Sondenpositionierung bei CRT. Die Implantation muss von einem erfahrenen chirurgischen Team vorgenommen werden, und erfordert aufgrund der Voroperationen und veränderten Anatomie meist eine (Re‑)Sternotomie [32]. Erste positive Erfahrungen mit Conduction System Pacing (d. h. Stimulation des His-Bündels oder des linken Tawara-Schenkels) und sondenlosen Schrittmachern bei EMAH liegen vor und machen diese zu Erfolg versprechenden Alternativen [33, 34].

#### Resynchronisationstherapie

Pathophysiologische und strukturelle Unterschiede bei EMAH und Herzinsuffizienz erschweren die Indikationsstellung und auch praktische Umsetzung einer CRT. Häufiger als ein Linksherzversagen besteht eine Insuffizienz eines systemischen rechten Ventrikels, eines subpulmonalen rechten Ventrikels oder eines Single-Ventrikels. Auch findet sich häufiger ein Rechtsschenkel- als ein Linksschenkelblock. Die besten Daten für eine Verbesserung der myokardialen Kontraktilität liegen für PatientInnen mit schrittmacherinduzierter Ventrikeldysfunktion bei Stimulation des subpulmonalen Ventrikels vor [8, 20]. Für EMAH mit antibradykarder Schrittmacherindikation, erwartetem Stimulationsanteil des Ventrikels > 40 % (evtl. schon > 20 %) bei einer EF ≤ 35 % sowie Personen mit systemischem rechtem Ventrikel wird generell eine CRT-Implantation empfohlen [8, 9]. Die Anatomie und Position des Koronarsinus in Relation zum Systemventrikel bestimmt die Möglichkeit zur intravenösen Sondenanlage, häufig ist jedoch die Implantation einer epikardialen ventrikulären Sonde erforderlich (z. B. Single-Ventrikel, Z. n. atrialer Switch-Operation, Versagen des subpulmonalen Ventrikels) [8, 9]. Generell wird angeraten, vor geplanten kardialen Operationen bei AHF die Wahrscheinlichkeit einer PSM-Indikation im Langzeitverlauf mitzubedenken. Im Rahmen der Operation könnten bereits epikardiale Sonden implantiert und bei Bedarf zu einem späteren Zeitpunkt an einen Schrittmacher angeschlossen werden [9].

### Implantierbarer Kardioverter-Defibrillator (ICD)

Eine klare Indikation zur ICD-Implantation bei EMAH besteht als Sekundärprophylaxe nach überlebtem Herz-Kreislauf-Stillstand aufgrund von Kammerflimmern oder hämodynamisch instabiler VT (I C) [8, 9, 18]. Auch nach einer anhaltenden VT wird eine ICD-Implantation empfohlen (I C) [8, 9, 18]. Indikationen zur Primärprophylaxe wurden aus den Studien und Empfehlungen erworbener Herzerkrankungen abgeleitet, eine Klasse IIa C Empfehlung gilt für Personen mit einer biventrikulären Zirkulation und eingeschränkter Funktion eines anatomisch linken Systemventrikels ≤ 35 % im NYHA Stadium II–III. [8, 9, 18]. Weiters soll eine ICD-Implantation bei EMAH mit Synkope und höhergradig eingeschränkter Funktion des Systemventrikels oder induzierbarem Kammerflimmern bzw. Kammertachykardie überlegt werden (IIa C) [8, 9, 18]. Ein signifikant erhöhtes Risiko für das Auftreten eines SCD besteht erfahrungsgemäß bei EMAH mit folgenden Vitien: TOF, d‑TGA nach atrialer Switch-Operation, ccTGA, Vitien mit linksseitigen Stenosen, zyanotische Herzfehler (Eisenmenger-Physiologie) und Ebstein-Anomalie [9, 18]. Neben Tachyarrhythmien können weiters Bradyarrhythmien, AV-Blockierungen und atriale Tachykardien zu plötzlichem Herztod bei EMAH führen. 20 % aller plötzlichen Todesfälle können jedoch nicht auf eine arrhythmogene Ursache zurückgeführt werden [9, 18]. Empfehlungen für eine primärprophylaktische ICD-Implantation beruhen bei diesen Vitien vor allem auf retrospektiven Studien und Expertenmeinungen. Bei Personen mit Single-Ventrikel oder systemischem rechtem Ventrikel mit höhergradig reduzierter Funktion kann bei Vorliegen eines weiteren Risikofaktors wie NYHA-Stadium II–III, QRS-Dauer > 140 ms, hochgradiger Insuffizienz der systemischen AV-Klappe oder nicht anhaltenden VTs eine ICD-Therapie erwogen werden (IIb C) [8, 9, 18]. Bei TOF gilt das Vorhandensein mehrerer Risikofaktoren wie nicht anhaltende VTs, eine eingeschränkte Linksventrikelfunktion, eine QRS-Dauer > 180 ms, ausgeprägte rechtsventrikuläre Narben oder induzierbare anhaltende Kammertachykardie als erhöhtes Risiko für einen SCD (IIa C) [8, 9, 18]. Überlegt werden kann eine ICD-Prophylaxe auch bei Personen auf der Warteliste zur Herztransplantation [8, 9, 18]. Atriale Tachykardien müssen adäquat mittels Ablation therapiert werden, da diese das SCD-Risiko signifikant erhöhen [8, 9, 18]. Zur Überbrückung der Zeit bis zu einer ICD-Implantation steht den PatientInnen der Wearable-Kardioverter-Defibrillator (WCD) zur Verfügung [35]. Mögliche Szenarien für die Anlage eines WCD sind fehlende Kapazitäten für eine baldige ICD-Implantation, eine Re-Evaluierung der ICD-Indikation oder eine baldige Herztransplantation (IIb C); nach ICD-Extraktion (v. a. wegen Infektionen) besteht derzeit eine Indikationsklasse IIa C für einen WCD [36].

Aufgrund des jungen Alters der EMAH und der limitierten Haltbarkeit der Komponenten von PSM und ICD-Systemen, sind im Laufe des Lebens rezidivierende Revisionen, Extraktionen und Neuimplantationen von Sonden und Generatorwechsel zu erwarten. Auch Sondenkomplikationen wie Endokarditiden, AV-Klappeninsuffizienzen und chronische Verschlüsse der venösen Zugänge können langfristig Probleme bereiten. Das Risiko ist für transvenöse ICD-Sonden nochmals signifikant höher als für PSM-Sonden [9, 32]. Subkutane ICD wurden bei EMAH erfolgreich eingesetzt, allerdings ist ein Screeningversagen aufgrund einer nicht ausreichenden Diskriminierung von R‑ und T‑Welle bei vorbestehenden Schenkelblockbildern häufiger. Zudem können das größere Aggregat in Relation zur oft kleinen Körpergröße sowie die (derzeit noch) fehlende Möglichkeit des antitachykarden Pacings limitierende Faktoren darstellen [9, 30, 37]. Sollte zusätzlich ein antibradykardes Pacing indiziert sein, besteht neben transvenösen Systemen auch die Option für eine epikardiale Elektrodenplatzierung [8, 9, 30]. Der extravaskuläre ICD mit substernaler ICD-Elektrode ermöglicht zwar antitachykardes Pacing bei einer dem transvenösen ICD identen Aggregatsgröße, allerdings stellt zum jetzigen Zeitpunkt eine vorausgegangene Sternotomie eine Kontraindikation zu dieser Therapie dar [38]. Vor einer ICD-Implantation sind sämtliche Informationen hinsichtlich kardialer Anatomie und venöser Zugänge (wie oben angegeben) einzuholen und zu evaluieren, ob auch eine Indikation zu einer Brady- oder Resynchronisationstherapie besteht [8, 9, 30].

### Arrhythmien in der Schwangerschaft

Arrhythmien sind die häufigsten kardialen Komplikationen in der Schwangerschaft [39–41]. Die hämodynamischen, strukturellen und hormonellen Veränderungen begünstigen sowohl Erstmanifestation sowie Rezidive von Arrhythmien. SVT sind hierbei häufiger als VT und Bradyarrhythmien [39, 41]. Eine gewissenhafte Abklärung von Symptomen möglicher Arrhythmien wie Palpitationen und Synkopen in der Schwangerschaft ist erforderlich. Tachy- und Bradyarrhythmien können und müssen in der Schwangerschaft adäquat therapiert werden. Idealerweise sollten bekannte Arrhythmien vor einer geplanten Schwangerschaft behandelt werden und stabil sein. Einen Überblick über die in der Schwangerschaft zulässigen Medikamente sowie Therapiealgorithmen sind in den entsprechenden Guidelines nachzulesen [39, 41].

## Vitienspezifische Aspekte von Arrhythmien bei EMAH

### 1. Vorhofseptumdefekte

Ungefähr 15 % aller kongenitaler Vitien sind atriale Septumdefekte (ASD). ASDs können isoliert oder als Teil komplexer Vitien vorkommen und sind das häufigste Beispiel für ein kongenitales Vitium mit Erstmanifestation im Erwachsenenalter. Durch die direkte Kommunikation auf Vorhofebene führen sie (bei Fehlen einer Pulmonalstenose und/oder einer pulmonalarteriellen Hypertonie) zu einem Links-Rechts-Shunt zwischen dem System- und dem Pulmonalkreislauf. Folge sind je nach Defektgröße, Druckunterschied zwischen rechtem und linkem Vorhof und Compliance der Ventrikel eine Zunahme des pulmonalen Blutflusses, eine rechtsventrikuläre Volumenbelastung und ein Remodeling des rechten Vorhofes mit konsekutiver Prädisposition für atriale Tachyarrhythmien (AT) [18, 42].

Die bei Erwachsenen typischen zur Diagnose führenden Beschwerden sind Einschränkung der Leistungsfähigkeit, Belastungsdyspnoe, AT, selten paradoxe systemische Embolien und manchmal rezidivierende pulmonale Infekte.

**Abb. 1:**
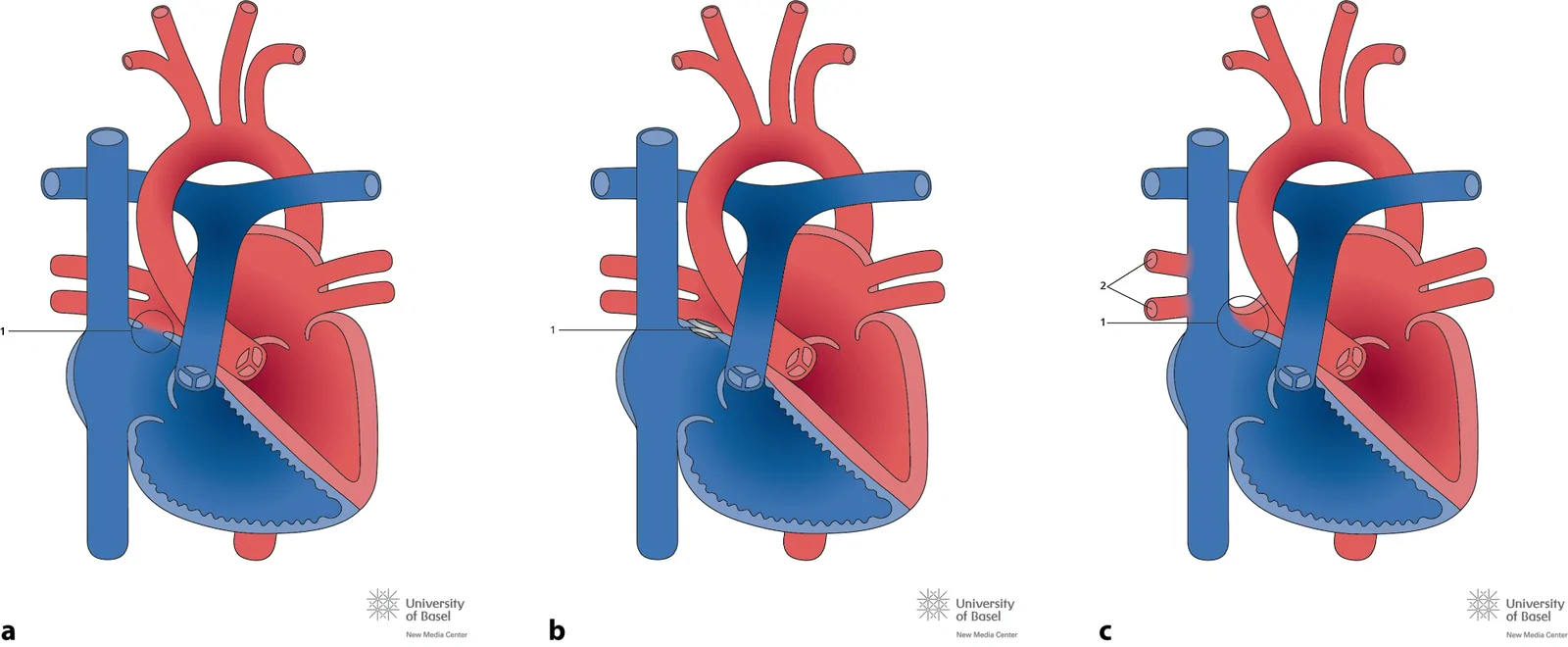
Vorhofseptumdefekte [89]. **a** Vorhofseptumdefekt vom Secundum-Typ: *1* Vorhofseptumdefekt. **b** Vorhofseptumdefekt vom Secundum-Typ nach interventionellem Verschluss: *1* Verschluss-Device, **c** superiorer Sinus-venosus-Defekt mit Fehlmündung der rechten oberen und mittleren Lungenvene in die V. cava superior: *1* Vorhofseptumdefekt, *2* rechte obere und mittlere Lungenvene münden in die V. cava superior

Abhängig von der Lokalisation des Defektes im Vorhofseptum werden 4 ASD-Typen unterschieden, welche je nach Defekttyp mit weiteren morphologischen Auffälligkeiten assoziiert sind. Der Secundum-ASD (ASD 2) im Bereich der Fossa ovalis und Umgebung ist die häufigste Form eines ASDs (ca. 80 %) und tritt bevorzugt bei Frauen auf (Abb. 1a und b; [42, 43]) Der Primum-ASD (ASD 1) ist ein partieller atrioventrikulärer Septumdefekt (AVSD), die spezifischen anatomische Details werden im entsprechenden Kapitel (s.unten) beschrieben. Sinus-venosus-Defekte liegen im Bereich der Einmündung der oberen und unteren Hohlvene in den rechten Vorhof (SVC häufiger als IVC) und sind in vielen Fällen mit Fehlmündung einer oder mehrerer Lungenvenen assoziiert (Abb. 1c). Die seltenste ASD-Variante ist der Koronarsinusdefekt, bei dem der Defekt im Dach des Sinus coronarius – und damit anatomisch im linken Vorhof – liegt. Der Shuntfluss in den rechten Vorhof erfolgt über das Ostium des Koronarsinus. Eine extreme Variante ist ein vollständig fehlender Sinus coronarius („unroofed coronary sinus“). Meist findet sich zusätzlich eine persistierende linke obere Hohlvene.

Jeder hämodynamisch wirksame ASD soll verschlossen werden: (cave: Bereits bestehende pulmonalarterielle Hypertonie, Linksherzinsuffizienz – s. entsprechende Leitlinie) [18], wobei im Erwachsenenalter der katheterinterventionelle Verschluss, sofern technisch möglich, bei ASDs in der Fossa ovalis (ASD 2) die bevorzugte Methode darstellt.

Auch für superiore Sinus-venosus-Defekte existieren bereits interventionelle Verschlussmöglichkeiten, die in ausgewählten Fällen zur Anwendung kommen können. Standard ist jedoch weiterhin die chirurgische Korrektur. Koronarsinusdefekte und partielle AVSDs können nur operativ verschlossen werden [18, 42].

#### Atriale Tachyarrhythmien

Das atriale Remodeling durch den Links-Rechts-Shunt begünstigt vor allem das Auftreten von Vorhofflattern und Vorhofflimmern [18, 42].

PatientInnen mit isoliertem ASD, die vor dem 25. Lebensjahr verschlossen wurden, haben eine normale Lebenserwartung (Mortalitätsbenefit) und langfristig ein niedriges Risiko für Spätkomplikationen inklusive AT [18, 42, 44]. Bis zum 40. Lebensjahr führt ein ASD-Verschluss zu einem atrialen Reverse-Remodeling und mittel- bis langfristig zu einer Abnahme der Häufigkeit aller AT [18, 42, 44]. In den ersten Wochen nach einem interventionellen ASD-Verschluss kann vorübergehend bis zur gänzlichen Endothelialisierung des Schirms die Häufigkeit von AT zunehmen [18, 42, 44]. Ein alleiniger ASD-Verschluss bei Erwachsenen über 40 Jahren beeinflusst die Häufigkeit der atrialen Rhythmusstörungen trotz verbesserter Hämodynamik und klinischer Symptomatik jedoch nicht mehr wesentlich [18, 42]. AT nach ASD-Verschluss sind am häufigsten rechtsatriales Isthmus-abhängiges Vorhofflattern, auch wenn das EKG postoperativ atypisch erscheinen kann [45].

#### Bradykardien

Beim operativen Verschluss von superioren Sinus-venosus-Defekten kann es zu Verletzungen des Sinusknotens und folglich zu einer Sinusknotendysfunktion und symptomatischen Bradykardien kommen [8, 9]. PatientInnen mit ASD I haben aufgrund der anatomischen Verlagerung des AV-Reizleitungssystems ein intrinsisch erhöhtes Risiko für AV-Blockierungen (s. unten) [18, 42].

#### Vorhofflimmern

Die Vorhofflimmerprävalenz bei Erwachsenen mit ASD, bei denen dieser noch nicht verschlossen wurde, ist deutlich höher als in der Allgemeinbevölkerung (ca. 25 % bei 35- bis 50-Jährigen und ca. 45 % bei über 50-Jährigen) [18, 42, 46, 47]. Eine Reduktion der Vorhofflimmerhäufigkeit nach alleinigem ASD-Verschluss konnte erwartungsgemäß nur bei PatientInnen mit paroxysmalem Vorhofflimmern gefunden werden [42, 46–50]. Im Langzeitverlauf war die Vorhofflimmerrate nach Pulmonalvenenisolation bei anschließendem interventionellem ASD-Verschluss niedriger [50] und wird daher empfohlen [42]. In der Studie wurden empirisch 3 rezidivfreie Monate vor dem ASD-Verschluss gefordert, um im Falle eines Rezidivs einen Wiederholungseingriff durchführen zu können, durchschnittlich erfolgte der Verschluss nach ca. 6 Monaten [50]. Nach einem interventionellen ASD-Verschluss ist eine transseptale Punktion in erfahrenen Zentren weiterhin möglich [51]. Die Erfolgsraten bei paroxysmalem Vorhofflimmern mittels Katheterablation und chirurgischer Ablation sind vergleichbar [46]. Wurde im Rahmen eines operativen ASD-Verschlusses eine biatriale chirurgische Ablation durchgeführt, war dies effektiver als eine alleinige rechtsatriale Ablation [46]. Es gibt keine ausreichenden Daten, die zeigen, dass eine Vorhofflimmerablation bei ASD-PatientInnen ohne dokumentiertes Vorhofflimmern indiziert ist.

### 2. Ventrikelseptumdefekte

**Abb. 2:**
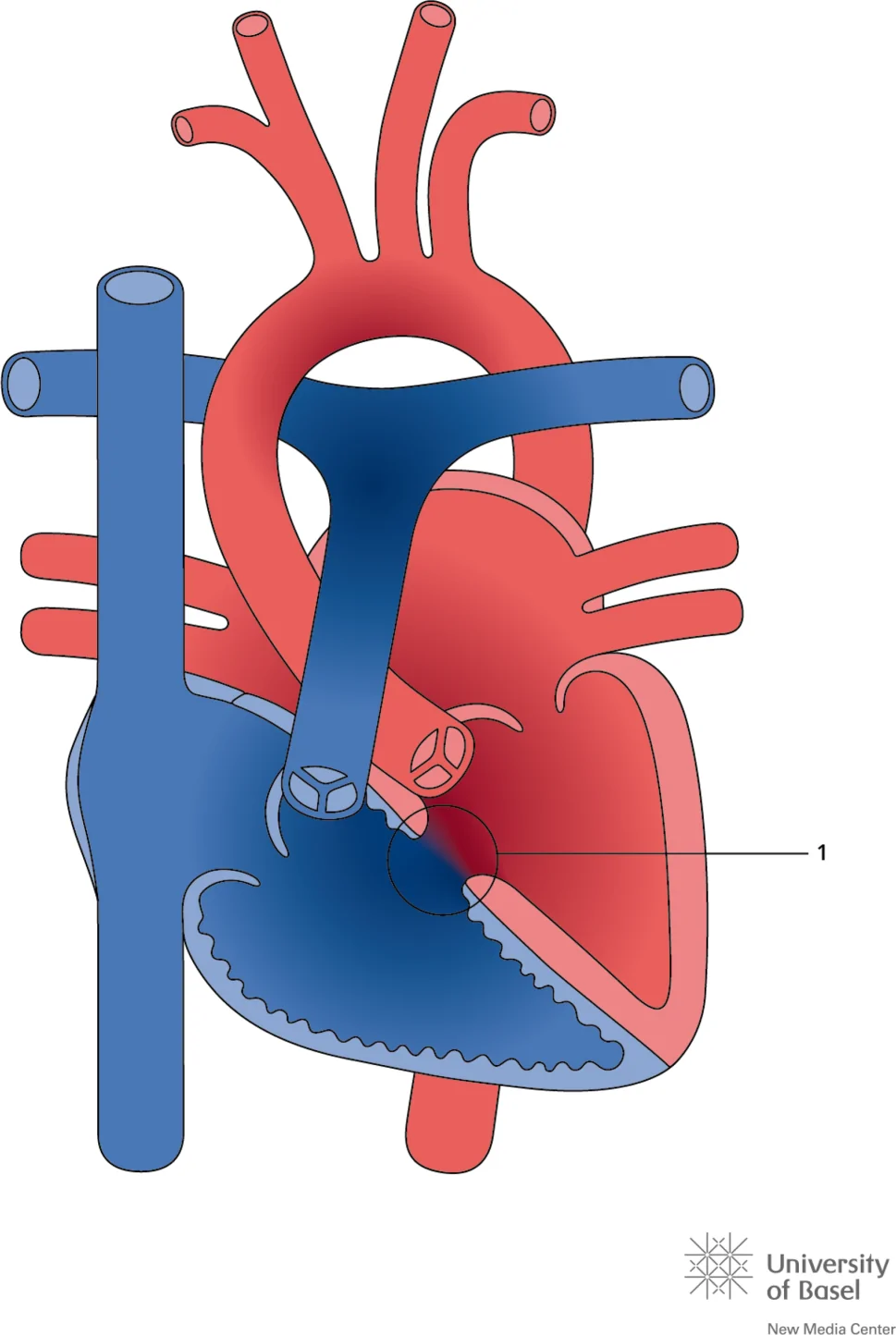
Perimembranöser Ventrikelseptumdefekt [89], *1* Ventrikelseptumdefekt

Ventrikelseptumdefekte (VSD) sind die häufigsten angeborenen Herzfehler und kommen isoliert sowie im Rahmen komplexer Fehlbildungen vor, manchmal finden sich auch multiple VSDs. Abhängig von der Lokalisation des Defektes im rechten Ventrikel werden perimembranöser VSD, muskulärer VSD, Outlet- oder subarterieller VSD und Inlet-VSD (auch atrioventrikulärer Septumdefekt – s. unten) unterschieden [18]. Der perimembranöse VSD ist mit rund 70–80 % die häufigste Form des Ventrikelseptumdefektes. Er liegt im membranösen Teil des Septums in unmittelbarer Nähe zu Aorten- und Trikuspidalklappe (Abb. 2). Der kompakte AV-Knoten findet sich üblicherweise in normaler Position, das HIS-Bündel verläuft inferoposterior des Unterrandes des VSD. Muskuläre VSD sind meist in ausreichendem Abstand zum Reizleitungssystem lokalisiert, ebenso Outlet-VSD [10]. Ein VSD führt zu einem systolischen Links-Rechts-Shunt, abhängig von der Defektgröße, dem Widerstand in Pulmonal- und Systemkreislauf sowie Stenosen des linken oder rechten Ausflusstraktes. Wird ein signifikanter Defekt nicht in den ersten Lebensjahren verschlossen kann sich bereits im Kindesalter eine fixierte pulmonalarterielle Hypertonie entwickeln. Weitere Komplikationen sind ein Aortenklappenprolaps mit konsekutiver Klappeninsuffizienz oder eine Obstruktion im rechten Ventrikel („double chambered right ventricle“). Therapeutisch erfolgt bei hämodynamisch signifikantem Shunt eine operative Korrektur mit Patchverschluss über einen transatrialen oder transventrikulären Zugang. Der interventionelle Verschluss ist aufgrund der Nähe zu AV-Knoten und Aortenklappe limitiert und bleibt ausgewählten Situationen vorbehalten. Besteht bereits eine fixierte pulmonalarterielle Hypertonie muss vor einem VSD Verschluss eine Hämodynamikmessung mit Bestimmung von Shuntvolumen und pulmonalem Gefäßwiderstand an einem erfahrenen Zentrum erfolgen und Nutzen und Risiko einer Operation sorgfältig abgewogen werden. Eine Eisenmenger-Reaktion stellt eine absolute Kontraindikation zu einem VSD Verschluss dar [18].

#### Arrhythmierisiko

Ein erhöhtes Risiko besteht vor allem für AV-Blockierungen (AVB) im Rahmen von operativen und interventionellen Verschlüssen von perimembranösen VSD [18]. Diese treten sofort perioperativ oder in der postoperativen Periode auf, sind im Langzeitverlauf dagegen selten [8, 9]. AVSD haben zusätzlich ein intrinsisch erhöhtes Risiko für AVB [8, 9].

Andere Arrhythmien: AT, ventrikuläre Tachykardien (VT), plötzlicher Herztod (SCD) sind seltener und oft Folge von möglichen Langzeitkomplikationen wie pulmonalarterieller Hypertonie, Ventrikeldysfunktion oder atrialem Remodeling. Weiters können VSD und Patches Substrate für ventrikuläre Reentrytachykardien darstellen, dies wurde am genauesten für PatientInnen mit TOF beschrieben (s. unten) [8, 9].

### 3. Atrioventrikuläre Septumdefekte

**Abb. 3:**
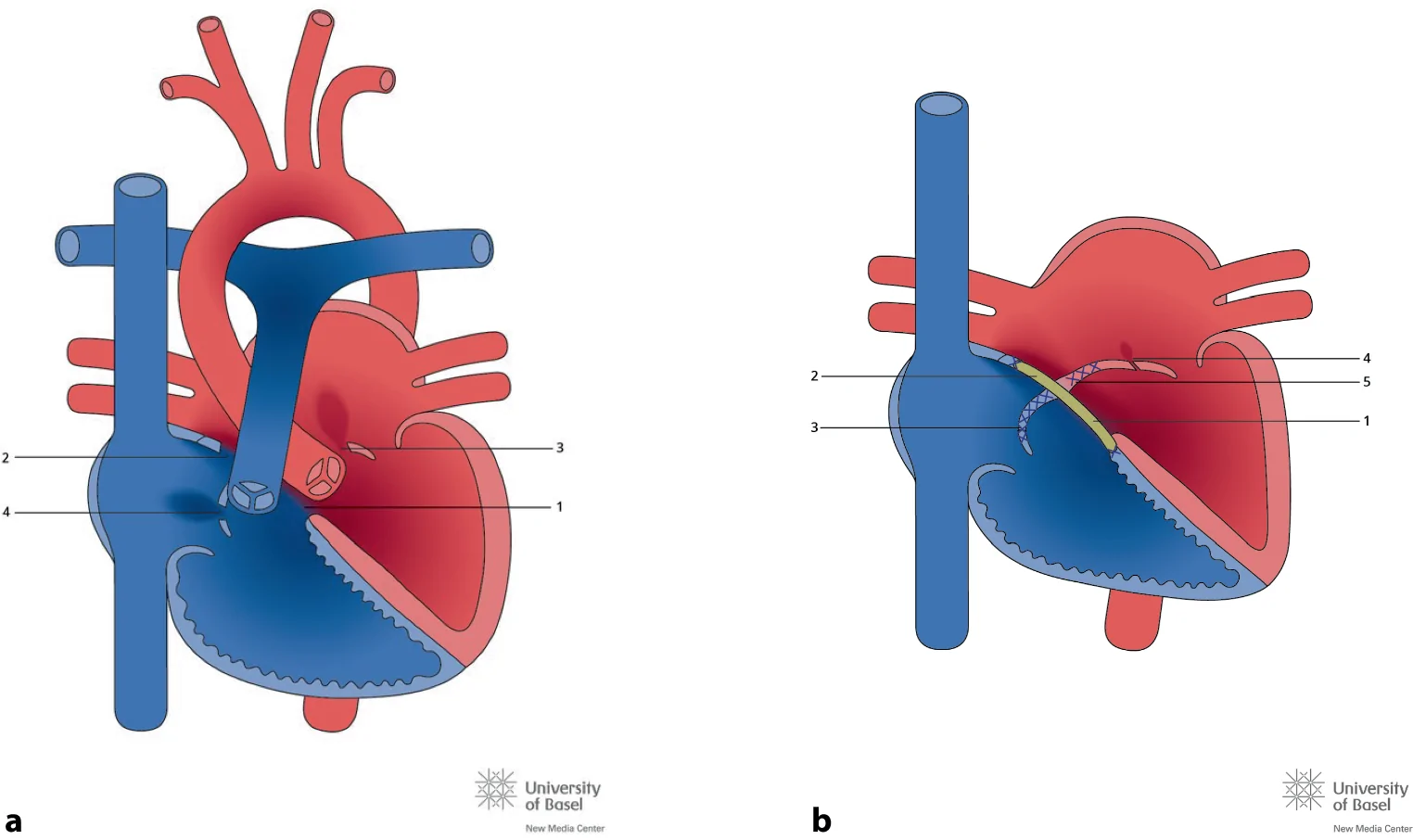
Kompletter atrioventrikulärer Septumdefekt [89]. **a** Präoperativ: *1* Ventrikelseptumdefekt vom Inlet-Typ, *2* Ostium Primum-Vorhofseptumdefekt (ASD 1), *3* Cleft der linken atrioventrikulären Klappe, *4* Cleft der rechten atrioventrikulären Klappe. **b** Postoperativ nach Korrekturoperation: *1* + *2* Patchverschluss des Vorhof- und Ventrikelseptumdefektes, *3* Rekonstruktion der rechten atrioventrikulären Klappe, *4* residuelles Cleft, *5* Rekonstruktion der linken atrioventrikulären Klappe

Der AVSD beschreibt ein Spektrum an Fehlbildungen, die den unteren Anteil des atrialen Septums, den Inlet-Bereich des ventrikulären Septums und die AV-Klappen betreffen. Je nachdem auf welchem Level eine Shuntmöglichkeit besteht, wird zwischen partiellem AVSD (atrialer oder ventrikulärer Shunt) und komplettem AVSD (atrialer und ventrikulär Shunt) unterschieden. Der partielle AVSD mit isoliertem atrialem Shunt wird auch als ASD 1 oder Primum-ASD bezeichnet. In allen Fällen findet sich eine gemeinsame AV-Junktion mit einer gemeinsamen AV-Klappe und unvollständiger bis fehlender Separation der Trikuspidal- und Mitralklappe (Abb. 3). Typisch beim partiellen AVSD ist eine trikuspide linksseitige AV-Klappe (Mitralklappe mit einem „Cleft“) mit konsekutiver Insuffizienz [42]. Das AV-Reizleitungssystem – sowohl der kompakte AV-Knoten als auch das HIS-Bündel – sind nach posterior und inferior in Richtung Koronarsinus verlagert [10, 18]. Die Therapie erfolgt chirurgisch durch Patchverschluss des atrialen und/oder ventrikulären Septumdefektes und Rekonstruktion der AV-Klappen [18, 42].

#### Atriale Tachyarrhythmien

Chronische Vorhofdilatation sowie AV-Klappenvitien prädisponieren für intraatriale Reentrytachykardien (IART) und später Vorhofflimmern. Postoperative Narben im Bereich des inferioren Vorhofseptums und der Klappennaht fördern die Entstehung von Makroreentry-Kreisen, die IART zugrunde liegen.

#### Bradykardien

Aufgrund der dislozierten Lage des AV-Knotens ist bereits präoperativ ein erhöhtes Risiko für AV-Blockierungen gegeben, diese können jedoch auch Jahre nach der Korrektur auftreten [8, 9].

#### Besonderheiten im Management

Aufgrund der verlagerten Position des AV-Reizleitungssystems besteht ein erhöhtes Risiko für AV-Blockierungen. Ablationen sollten ausschließlich in spezialisierten Zentren erfolgen [8, 9].

### 4. Konotrunkale Fehlbildungen

Konotrunkale Fehlbildungen umfassen eine Gruppe von Anomalien, die durch komplexe morphologische Veränderungen der proximalen Ausflussbahnen des Herzens gekennzeichnet sind. Dazu zählen die Fallot-Tetralogie (TOF), die komplette Transposition der großen Arterien (d-TGA), der „double outlet right ventricle“ (DORV) und der Truncus arteriosus communis [52]. Diese Vitien zählen zu den zyanotischen Herzfehlern, und eine chirurgische Korrektur wird üblicherweise frühzeitig im Kindesalter vorgenommen. Die Operation erfordert meist 1. einen VSD-Patchverschluss mit Tunnelung des linken Ventrikels zum näher gelegenen arteriellen Gefäß (meist zur Aorta) und 2. die Rekonstruktion des rechtsventrikulären Ausflusstraktes (RVOT) zur Verbindung des rechten Ventrikels mit den Pulmonalarterien durch einen oder mehrere folgender chirurgischer Schritte: Patcherweiterung des RVOT, Valvulotomie der Pulmonalklappe, Resektion von subvalvulär stenosierenden muskulären Strukturen, Conduitimplantation vom rechten Ventrikel zu den Pulmonalarterien und/oder Switch-Operation der großen Arterien [53].

Im Langzeitverlauf besteht ein erhöhtes Risiko für Arrhythmien sowohl für atriale als auch für ventrikuläre Rhythmusstörungen, wobei die Art und das Auftreten der Rhythmusstörungen von der ursprünglichen Anatomie, den chirurgischen Korrekturen sowie der postoperativen Hämodynamik abhängen. Bei Neudiagnose von Arrhythmien muss unbedingt eine Abklärung hinsichtlich möglicher struktureller Komplikationen erfolgen. Bei PatientInnen mit diesen Vitien liegen oft eine Degeneration implantierter Conduits, eine Trikuspidalklappeninsuffizienz, Stenosen im linksventrikulären Ausflusstrakt (LVOT), residuelle Shunts u. a. vor. Erwartungsgemäß sind im Langzeitverlauf auch nach erfolgter Korrekturoperation im Kindesalter Re-Operationen bzw. Interventionen notwendig [18]. Lage und der Verlauf des AV-Reizleitungssystems sind abhängig von der Art des VSD (s. Kapitel VSD) [10, 54]. Sowohl AT als auch VT und SCD sind häufig [18, 55]. Die Mechanismen der VTs bei PatientInnen mit TOF gehören zu den am besten charakterisierten im Spektrum der angeborenen Herzfehler (AHF) [55].

#### a. Fallot-Tetralogie

**Abb. 4:**
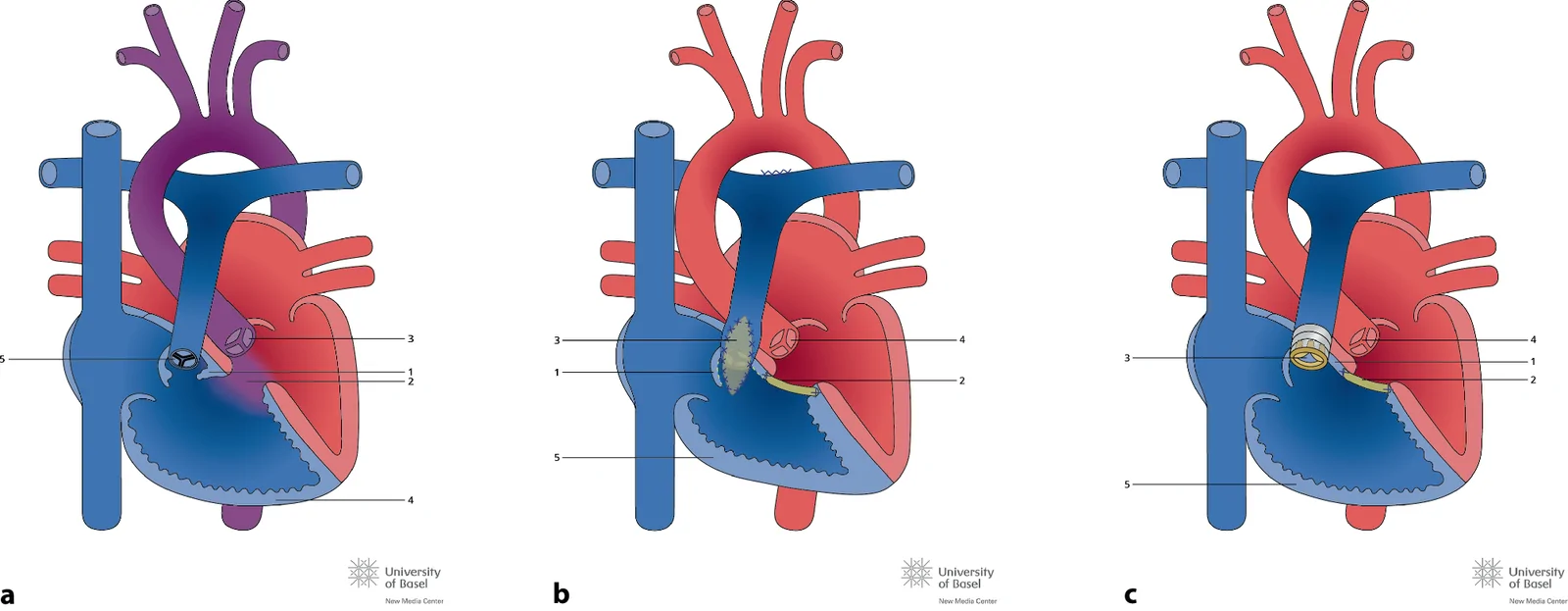
Fallot-Tetralogie [89]. **a** Fallot-Tetralogie mit Pulmonalstenose: *1* muskuläre infundibuläre Stenose, *2* Ventrikelseptumdefekt, *3* überreitende Aorta, *4* rechtsventrikuläre Hypertrophie, *5* Pulmonalklappenstenose. **b** Postoperativ nach Korrekturoperation mit transanulärem Patch: *1* Resektion der muskulären infundibulären Stenose, *2* Patchverschluss des Ventrikelseptumdefektes, *3* transanulärer Patch, *4* überreitende Aorta, *5* rechtsventrikuläre Hypertrophie. **c** Postoperativ nach Implantation einer Pulmonalklappenprothese: *1* Resektion der muskulären infundibulären Stenose, *2* Patchverschluss des Ventrikelseptumdefektes, *3* biologische Pulmonalklappenprothese, *4* überreitende Aorta, *5* rechtsventrikuläre Hypertrophie

Die TOF ist der häufigste zyanotische Herzfehler, der etwa 4,5 % aller angeborenen Herzfehler ausmacht [43]. Die 4 pathognomonischen Merkmale umfassen eine infundibuläre Obstruktion des RVOT, einen VSD, eine Dextroposition (Überreiten nach rechts über dem Ventrikelseptum) der Aorta und eine rechtsventrikuläre Hypertrophie (Abb. 4).

Der AV-Knoten liegt bei PatientInnen mit TOF in der Regel in normaler Position; der Verlauf des His-Bündels ist abhängig vom Typ des VSD, der meist ein perimembranöser VSD ist, jedoch kommen auch muskuläre und subarterielle VSD vor (s. Kapitel VSD).

Die chirurgische Korrektur, die in der Regel im Kleinkindalter erfolgt, umfasst den VSD-Patchverschluss sowie die Beseitigung der RVOT-Obstruktion. Bei sehr kleinem Durchmesser des Pulmonalklappenrings wird der Patch zur Erweiterung des RVOT über den Pulmonalklappenanulus hinaus verlängert (transanulärer Patch), das begünstigt eine freie Pulmonalklappeninsuffizienz [55]. Im Langzeitverlauf sind Reoperationen häufig notwendig, vor allem wenn die Volumenbelastung durch die Pulmonalklappeninsuffizienz zu einer signifikanten Dilatation des rechten Ventrikels führt und die Implantation einer Pulmonalklappenprothese indiziert ist [18]. Bei vielen älteren Erwachsenen mit TOF wurde die Korrekturoperation über eine Ventrikulotomie des RVOT durchgeführt. Später wurde dies durch einen transatrialen und transpulmonalen Zugang ersetzt [55, 56].

##### Atriale Tachyarrhythmien

AT, IART und mit zunehmendem Alter (ab 45 Jahren) auch Vorhofflimmern sind häufig. Die Substrate sind Inzisionsnarben nach Atriotomie, ASD-Patches sowie Dilatation des rechten und linken Vorhofes [57]. AT sind wiederum ein Risikofaktor für das Auftreten von VT [58].

##### Ventrikuläre Arrhythmien

PatientInnen mit TOF haben ein signifikant erhöhtes Risiko für VT und SCD [55]. Die Prävalenz anhaltender VT nach 30 Jahren Nachbeobachtung wird auf 14 % geschätzt [57]. Die Inzidenz von SCD beträgt in etwa 0,15 % pro Jahr, ist somit 50- bis 200-mal höher als in der Allgemeinbevölkerung [59] und hängt mit der hohen Prävalenz anhaltender VT zusammen [57]. Die Mechanismen der VT sind sehr genau charakterisiert, insofern existieren hier eindeutigere Empfehlungen hinsichtlich Primär‑, Sekundärprävention und Ablation von ventrikulären Arrhythmien als bei anderen AHF [55]. Monomorphe VT sind meist Makro-Reentrytachykardien, die über definierte Isthmen rund um Narbenareale und Patches im rechten Ventrikel kreisen (s. unten). Häufig verbleibende hämodynamische Residuen mit chronischer Volums- und/oder Druckbelastung des rechten Ventrikels können langfristig zu einer ventrikulären Dilatation, Dysfunktion und einer diffusen myokardialen Fibrose führen. Dies wiederum kann das Auftreten schlechter charakterisierter polymorpher VT und Kammerflimmern begünstigen [55]. Bei der Risikostratifizierung und Behandlung von VT bei TOF-Patienten sind diese unterschiedlichen Arrhythmiesubstrate und -mechanismen zu beachten.

##### ICD-Implantation

ICD sind zur Sekundärprävention nach einem überlebten SCD oder bei anhaltender VT (definiert durch eine Dauer > 30 s oder hämodynamischer Instabilität) indiziert (I C) [8, 9, 18]. Eine ICD-Primärprävention soll bei Vorliegen mehrerer Risikofaktoren überlegt werden. Zu diesen zählen eine linksventrikuläre Dysfunktion, symptomatische nicht anhaltende ventrikuläre Tachykardien, ausgeprägte rechtsventrikuläre Narben in der Magnetresonanztomographie, eine QRS-Dauer von über 180 ms sowie eine induzierbare anhaltende ventrikuläre Tachykardie bei programmierter elektrischer Stimulation (IIa C) [8, 9, 18]. Eine weitere Indikation ist eine unerklärliche Synkope mit vermutlich arrhythmischer Ursache bei höhergradiger ventrikulärer Dysfunktion oder induzierbarer VT (IIa C) [8, 9, 18]. Verschiedene Risikoscores zur Prädiktion von TOF-PatientInnen mit hohem Risko für SCD und ventrikuläre Arrhythmien wurden publiziert, allerdings sind diese sehr unterschiedlich und zum jetzigen Zeitpunkt nicht geeignet, jene PatientInnen zu identifizieren, die von einer primärprophylaktischen ICD-Implantation profitieren [60, 61].

##### Programmierte Ventrikelstimulation und elektroanatomisches Mapping

Der häufigste Mechanismus für VT bei TOF sind Makroreentry-Kreise innerhalb des rechten Ventrikels, obwohl gelegentlich auch fokale VT und VT aus dem linken Ventrikel beobachtet werden [62]. Makroreentry-Kreise entstehen in Zonen mit fixem oder funktionellem Block durch kritische Isthmen zwischen Narbenarealen mit Fibrose nach chirurgischer Inzision, Prothesenmaterialien und Patches sowie den Klappenanuli der Trikuspidal- und Pulmonalklappe. Somit wurden bei TOF-PatientInnen 4 kritische VT-Isthmen identifiziert [62]: (1) Isthmus 1 ist begrenzt durch den Trikuspidalklappenanulus und die Inzisionsnarbe an der freien Wand des rechten Ventrikels, den RVOT-Patch oder den transanulären Patch, (2) Isthmus 2 durch den Pulmonalklappenanulus und die Inzisionsnarbe an der freien Wand des rechten Ventrikels oder den RVOT-Patch (nur bei 25 % vorhanden), (3) Isthmus 3 durch den Pulmonalklappenanulus und den VSD-Patch und (4) Isthmus 4 bei PatientInnen mit muskulösem VSD durch den VSD-Patch und den Trikuspidalklappenanulus (nur bei 10 % vorhanden). Die Isthmen 1 und 3 sind die häufigsten kritischen Isthmen für VT bei TOF-PatientInnen. Isthmus 3 ist dabei immer vorhanden, unabhängig davon, ob die Operation mittels Ventrikulotomie oder transatrial durchgeführt wurde [62].

##### Katheterablation

Eine Katheterablation kann bei symptomatischer VT zur Verringerung des Risikos adäquater ICD-Therapien durchgeführt werden. Weiters sollte überlegt werden, vor einem geplanten chirurgischen Pulmonalklappenersatz oder perkutaner Pulmonalklappenimplantation mittels Ventrikelstimulation eventuell vorhandene zukünftige Arrhythmiesubstrate zu identifizieren und zu abladieren, bevor sie nach Einbringung des Prothesenmaterials möglicherweise nicht mehr zugänglich sind [8, 55]. Bei ausgewählten PatientInnen mit hämodynamisch stabiler VT und einem einzelnen identifizierten Isthmus kann eventuell nach Ablation dieses Isthmuses mit einer ICD-Implantation zugewartet werden, wenn keine ventrikuläre Dilatation, Dysfunktion oder ausgeprägte Narbenbildung besteht, da in dieser selektionierten PatientInnengruppe die Rezidivrate sehr niedrig ist [55]. Zuletzt konnte auch gezeigt werden, dass durch eine elektrophysiologische Untersuchung mit korrekter Identifizierung und anschließender prophylaktischer Ablation von VT-Isthmen die primärprophylaktische ICD-Implantationsrate bei TOF-PatientInnen signifikant reduziert werden konnte [63].

Als Endpunkt für die erfolgreiche Ablation müssen alle vorhandenen VT-Isthmen blockiert sein. Dies wird durch den Nachweis eines bidirektionalen Blocks über die Linien sowie die Nichtinduzierbarkeit von VT am Ende der Prozedur bestätigt [55].

#### b. „Double outlet right ventricle“

Bei PatientInnen mit „double outlet right ventricle“ entspringen beide großen Arterien aus dem RV und machen 1,3 % aller angeborenen Herzfehler aus [43]. Die anatomische Heterogenität ist jedoch sehr groß. Abhängig unter anderem von der Art, Größe und Anzahl der VSD, der Position der großen Arterien und des Konusseptums, subarteriellen Stenosen und der Ventrikelvolumina sind unterschiedlichste chirurgische Strategien zur Korrektur erforderlich [54]. Die ursprünglichen Anatomie und Art der Korrektur beeinflusst Prognose, Komplikations- und Arrhythmierisiko im Langzeitverlauf [55].

#### c. Truncus arteriosus communis

Der Truncus arteriosus communis ist selten. Ein gemeinsamer arterieller Stamm entspringt aus dem Ventrikel mit einer arteriellen Klappe, aus dem die Koronararterien, die Aorta und die Pulmonalarterie abgehen. In den meisten Fällen ist ein subarterieller VSD vorhanden. Die Korrekturoperation erfordert neben dem VSD-Patch-Verschluss und der Herstellung einer Verbindung zwischen rechtem Ventrikel und Pulmonalarterie (meist Implantation eines Conduits) häufig auch die Sanierung von weiteren assoziierten Läsionen wie Trunkusklappenanomalien, Trikuspidalklappeninsuffizienz oder eines unterbrochenen Aortenbogens. Rezidivierende Reoperationen sind im Laufe des Lebens erforderlich. Das Risiko für AT und VT wird als ähnlich wie bei PatientInnen mit TOF angesehen [64].

#### d. (Komplette) Transposition der großen Arterien (d-TGA)

**Abb. 5:**
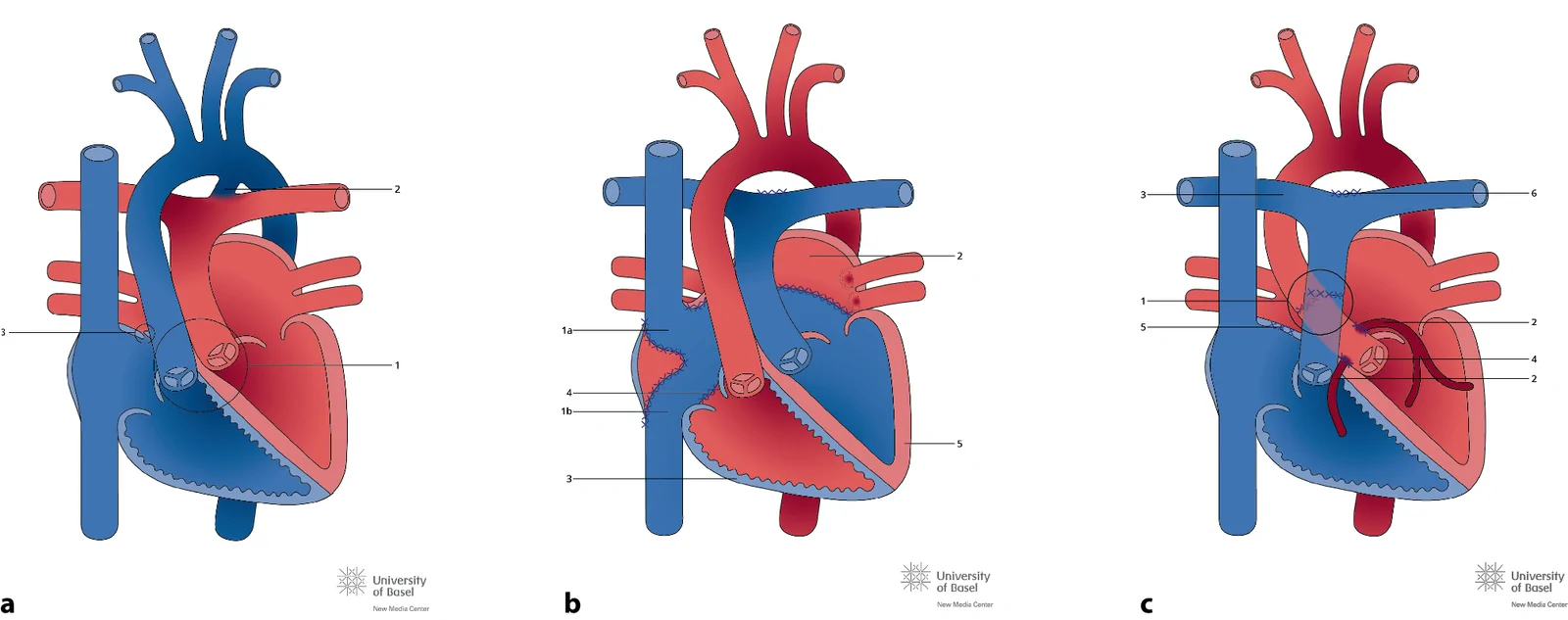
Komplette Transposition der großen Arterien [89]. **a** Präoperativ: *1* d-Transposition der großen Arterien, *2* persistierender Ductus arteriosus Botalli, *3* offenes Foramen ovale. **b** Postoperativ nach atrialer Switch-Operation: *1a* superiorer und *1b* inferiorer systemischer venöser Baffle, *2* pulmonalvenöser Vorhof, *3* subaortaler systemischer rechter Ventrikel, *4* systemische Trikuspidalklappe, *5* subpulmonaler linker Ventrikel. **c** Postoperativ nach arterieller Switch-Operation: *1* arterieller Switch, *2* Reimplantation der Koronararterien, *3* LeCompte-Manöver, *4* Neo-Aortenklappe, *5* Verschluss des atrialen Shunts, *6* Ligatur des persistierenden Ductus arteriosus

Die d‑TGA macht 4 % aller angeborenen Herzfehler aus [43]. Die Aorta entspringt aus dem rechten Ventrikel, während die Pulmonalarterie vom linken Ventrikel abgeht (Abb. 5). Häufig assoziierte Läsionen sind ein VSD, eine linksventrikuläre Ausflusstraktstenose oder eine Aortenisthmusstenose. Ohne Korrekturoperation ist ein Überleben in der Neugeborenenperiode unwahrscheinlich. Bis in die frühen 1990er-Jahre wurde die d‑TGA mittels atrialer Switch-Operation (Vorhofumkehroperation) palliiert. Seitdem wird hauptsächlich eine arterielle Switch-Operation durchgeführt. In Fällen mit hochgradiger Ausflusstraktstenose können auch ein VSD-Verschluss und die Implantation eines Conduits vom rechten Ventrikel zu den Pulmonalarterien erforderlich sein (Rastelli-Operation) [18].

Die Art der Korrekturoperation bestimmt bei PatientInnen mit d‑TGA die Risiken für Komplikationen und die Langzeitprognose [18].

##### i) Atriale Switch-Operation

Bei der atrialen Switch-Operation (Senning- oder Mustard-Operation) werden nach Resektion des Vorhofseptums Patches so in die Vorhöfe eingenäht, dass der pulmonalvenöse Rückfluss in Richtung Trikuspidalklappe (PV-Baffle) und der systemvenöse Rückfluss in Richtung Mitralklappe (SVC und IVC-Baffle) umgeleitet werden. Der morphologisch rechte Ventrikel liegt weiterhin subaortal und übernimmt die Funktion des Systemventrikels [65].

##### Bradykardien

Durch ausgedehnte Inzisionen und Nahtlinien im Vorhof können die Sinusknotenfunktion und das atriale Reizleitungssystem beschädigt sein, langfristig besteht ein erhöhtes Risiko für Bradykardien und chronotrope Inkompetenz [8, 9, 18]. Eine Schrittmacherimplantation über venöse Zugänge ist möglich, hierfür ist die Kenntnis der Anatomie zur korrekten Elektrodenplatzierung erforderlich. Im Falle der Notwendigkeit einer passageren Schrittmachertherapie ist es hilfreich zu wissen, dass die Schrittmachersonde im morphologisch linken Ventrikel platziert wird, der subpulmonal liegt. Eine Stenose des SVC-Baffles kann das Vorschieben der Schrittmachersonde erschweren und sollte vor permanenter Schrittmacher- oder ICD-Implantation ausgeschlossen werden [18].

##### Atriale Tachyarrhythmien

Die chirurgischen Nahtlinien stellen auch die Grundlage für IART dar [66], welche bereits im zweiten Lebensjahrzehnt auftreten können [67]. Makroreentrytachykardien kommen häufiger vor, fokale bzw. lokalisierte Reentrytachykardien sind seltener. Makroreentrytachykardien umfassen in der Regel ein cavotrikuspidales Isthmus-abhängiges oder ein inzisionsbedingtes Flattern [68]. Eine konsequente und rasche Therapie von AT ist erforderlich, da diese zu einer hämodynamischen Instabilität und auch zu VT führen können (s. unten). Bei der Gabe von antiarrhythmischen Medikamenten kann es zu einer Verlangsamung der atrialen Zykluslänge und bei schnell leitendem AV-Knoten zu einer 1:1-Überleitung kommen. Sowohl bei einer elektrischen Kardioversion als auch bei Verabreichung einer antiarrhythmischen Medikation muss mit der Möglichkeit einer gleichzeitig vorliegenden Sinusknotendysfunktion gerechnet werden, und Möglichkeiten zur vorübergehenden Schrittmacherstimulation müssen vorbereitet werden. Eine Betablockermedikation bei AT kann eventuell das Risiko für das Auftreten von VT reduzieren [69]. Eine Ablation stellt aufgrund der komplexen atrialen Anatomie eine technische Herausforderung dar. Sehr häufig findet sich ein Reentry um die Trikuspidalklappe im pulmonalvenösen Anteil des Vorhofs, der nur durch Baffle-Punktion oder retrograd über die Aorta erreicht werden kann. Die Rezidivrate ist hoch [68].

##### Ventrikuläre Arrhythmien

d‑TGA nach atrialer Switch-Operation gehört zu den angeborenen Herzfehlern mit dem höchsten SCD-Risiko, und SCD ist neben Herzinsuffizienz die häufigste Todesursache bei diesen PatientInnen (28,0–42,8 %) [18, 70]. In 73–83 % der SCD-Ereignisse werden ventrikuläre Arrhythmien vermutet [71]. Die meisten VT sind polymorphe VT oder Kammerflimmern [72]. Ursächlich werden einerseits primäre VT bei Herzinsuffizienz des rechten Systemventrikels und andererseits myokardiale Ischämien auf Basis einer Minderperfusion des druckbelasteten und hypertrophierten rechten Systemventrikels bei hohen atrialen Frequenzen (AT, Sinustachykardie) diskutiert [9, 69]; 80 % der SCD-Ereignisse treten bei körperlicher Belastung auf [69, 71]. Die Identifizierung geeigneter KandidatInnen für eine primärprophylaktische ICD-Implantation bleibt eine Herausforderung. Es besteht eine schwache Empfehlung für PatientInnen mit eingeschränkter systemischer rechtsventrikulärer Auswurffraktion (< 35 %), wenn zusätzliche Risikofaktoren wie nicht anhaltende VT, NYHA-Funktionsklasse II oder III, eine QRS-Dauer von ≥ 140 ms, unklare vermutlich arrhythmische Synkope oder eine schwere systemische AV-Klappeninsuffizienz vorhanden sind (IIb C) [8, 18]. Es wurden nur sehr wenige Fälle von VT-Ablationen veröffentlicht, bei denen das zugrunde liegende Substrat im rechten Ventrikel diffus zu sein scheint [68]. Eine Ventrikelstimulation zur Risikostratifizierung wird bei diesen PatientInnen nicht angeraten [9].

##### ii) Arterielle Switch-Operation

Seit den 1980er- und 1990er-Jahren werden PatientInnen mit d‑TGA mittels arterieller Switch-Operation (Jatene-Operation) korrigiert. Die Pulmonalarterie und die Aorta werden jeweils oberhalb des Sinus durchtrennt, die Pulmonalarterie nach anterior und die Aorta nach posterior umpositioniert, wodurch eine anatomisch korrekte ventrikuloarterielle Verbindung hergestellt wird. Hierbei müssen auch die Koronararterien, die zuvor mittels Buttons exzidiert werden, mit der Aorta transferiert und neu inseriert werden [65]. Intrakardiale Korrekturen sind nur im Falle begleitender Defekte, wie z. B. bei VSD, erforderlich. Arrhythmien treten deutlich seltener auf, sind jedoch insbesondere mit Koronaranomalien oder -komplikationen nach Insertion der Koronararterien assoziiert, die zu einer myokardialen Ischämie und konsekutiv zu VT und SCD führen können [73, 74].

##### iii) Rastelli-Operation

d‑TGA mit großem VSD und subpulmonaler Stenose werden häufig durch eine Rastelli-Operation korrigiert. Diese umfasst einen Patchverschluss des VSD und Implantation eines Conduits vom rechten Ventrikel zu den Pulmonalarterien [65]. Im Langzeitverlauf sind rezidivierende Reoperationen/-interventionen hauptsächlich zum Wechseln des Conduits notwendig. Atriale und ventrikuläre Arrhythmien sind häufig, und strukturelle Komplikationen, wie z. B. ein degenerativ veränderter Conduit, eine Stenose im LVOT oder ein signifikanter residualer Shunt, müssen ausgeschlossen werden [18, 65].

### 5. PatientInnen mit Fontan-Zirkulation

**Abb. 6:**
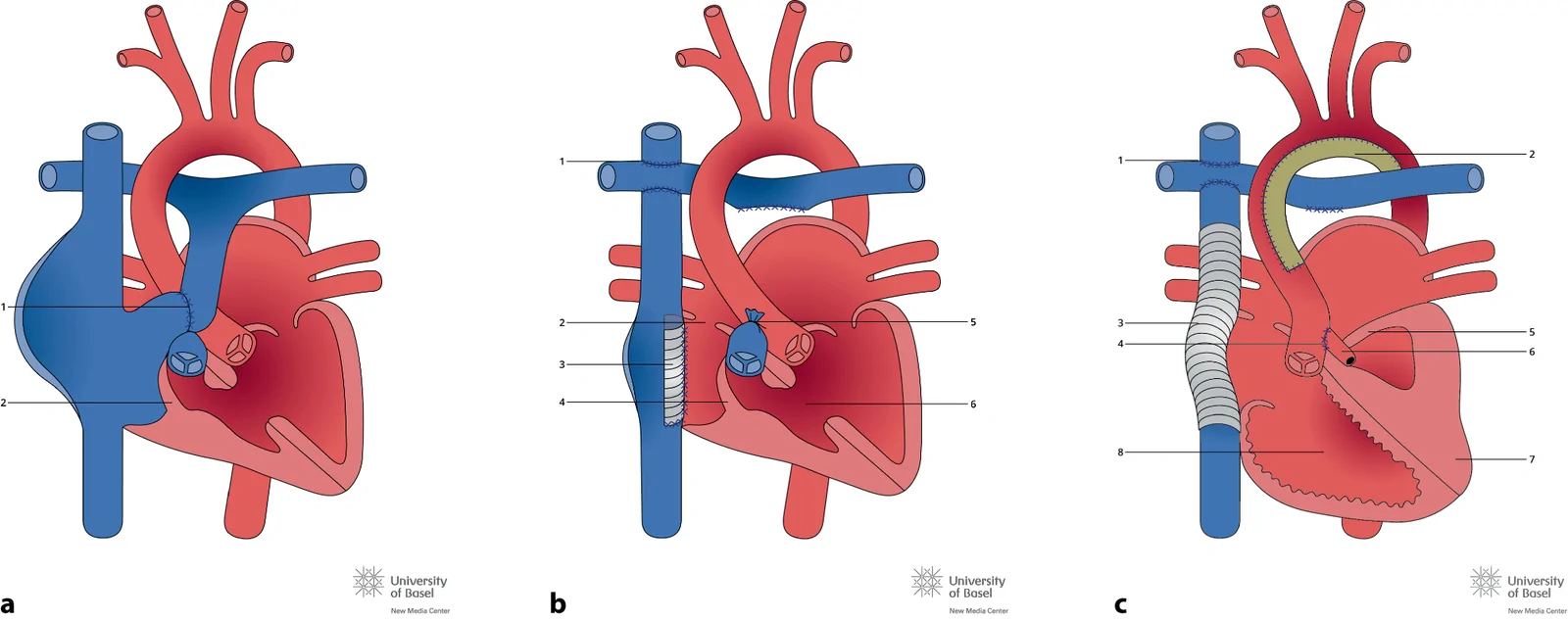
Univentrikuläre Palliation mit einem Fontan-Kreislauf [89]. **a** Atriopulmonale Fontan-Operation: *1* atriopulmonale Anastomose, *2* Trikuspidalklappenatresie. **b** Totale cavopulmonale Anastomose mit lateralem Tunnel: *1* totale cavopulmonale Anastomose (Glenn‑V. cava superior mit der rechten Pulmonalarterie), *2* Vorhofseptumdefekt, *3* lateraler Tunnel, *4* Trikuspidalklappenatresie, *5* abgesetzte Pulmonalarterie, *6* Ventrikelseptumdefekt. **c** Totale cavopulmonale Anastomose mit extrakardialem Conduit: *1* totale cavopulmonale Anastomose (Glenn‑V. cava superior mit der rechten Pulmonalarterie), *2* Patcherweiterung der Aorta, *3* extrakardialer Conduit, *4* Anastomose der Pulmonalarterie mit der hypoplastischen Aorta, *5* Mitralklappenatresie, *6* Aortenklappenatresie, *7* hypoplastischer linker Ventrikel, *8* rechter Systemventrikel

Die Gemeinsamkeit von PatientInnen mit einer Fontan-Zirkulation ist die spezifische Physiologie nach einer Serie von chirurgischen Eingriffen, welche Kindern, die mit einem univentrikulären Herzfehler geboren wurden, das Überleben ermöglichen. Wenn nur eine Herzkammer kräftig genug ausgebildet ist, um ein ausreichendes Schlagvolumen zu generieren, wird durch direkten Anschluss des systemvenösen Rückflusses an die Lungenzirkulation eine serielle Schaltung des Lungen- und des Körperkreislaufes geschaffen (Abb. 6). Der singuläre Ventrikel liegt subaortal und pumpt das Blut in den Körperkreislauf, während der Lungenkreislauf keine eigene Pumpe besitzt. Die treibende Kraft der Lungenperfusion ist der Druckgradient zwischen den großen Systemvenen und dem linken Vorhof. Die Fontan-Operation ist die letzte dieser Serie von Operationen und wurde seit der Erstbeschreibung 1971 mehrfach modifiziert. Während früher eine direkte Anastomose des rechten Vorhofs mit der Pulmonalarterie durchgeführt wurde (atriopulmonaler Fontan), sind die moderneren Versionen – auch als totale cavopulmonale Anastomose (TCPC) bezeichnet – entweder eine Patchabtrennung des systemvenösen Flusses innerhalb des rechten Vorhofes (lateraler Tunnel Fontan) oder ein extrakardialer Goretex-Conduit (extrakardialer Fontan), der die IVC mit der Pulmonalarterie verbindet. Zuvor muss bereits die SVC ebenfalls mit der Pulmonalarterie anastomosiert worden sein (Glenn-Operation) [18, 75, 76]. Das langfristige Risiko für Arrhythmien wird einerseits von der Anatomie des vorliegenden Herzfehlers, aber auch von der Art der operativen Palliation bzw. der Fontan-Modifikation bestimmt. Univentrikuläre Herzfehler umfassen anatomische Varianten mit Atresie einer AV-Verbindung (z. B. Trikuspidalatresie), einer komplett hypoplastischen Herzkammer (z. B. hypoplastisches Linksherzsyndrom), unbalancierte AVSD, „double inlet ventricle“ und Heterotaxiesyndrome [18, 77].

#### Atriale Tachyarrhythmien

Atriale Tachyarrhythmien können bei allen Fontan-Typen auftreten, am häufigsten jedoch bei PatientInnen mit einem atriopulmonalen Fontan aufgrund der über die Jahre zunehmenden Drucksteigerung und Dilatation des rechten Vorhofes, gefolgt von PatientInnen mit einem lateralen Tunnel-Fontan wegen der Vernarbungen entlang der Nahtlinien des Patches. Am wenigsten oft und zu einem späteren Zeitpunkt treten AT bei PatientInnen mit einem extrakardialen Conduit auf [75, 78, 79]. Sowohl IART, typisches isthmusabhängiges Vorhofflattern und auch Vorhofflimmern sind häufig, und mehrere Arrhythmiesubstrate können simultan vorhanden sein, während fokale AT seltener vorkommen [78]. Aufgrund der oft längeren Zykluslänge der AT kann es zu einer 1:1-Überleitung kommen, aber auch eine 2:1-Überleitung wird aufgrund der speziellen Physiologie oft hämodynamisch schlecht toleriert [7]. Das Risiko für Thromboembolien bei Auftreten von AT ist sehr hoch [7, 75]. AT treten ab 10 Jahren nach der Fontan-Operation bereits im jugendlichen und jungen Erwachsenenalter auf, und das Risiko steigt mit zunehmendem Alter [75, 80]. Die genaue Inzidenz bei PatientInnen mit den moderneren TCPC-Fontan ist aufgrund der derzeit limitierten Langzeitdaten noch nicht bekannt. Sicher ist, dass das Rezidivrisiko der AT sehr hoch ist und antiarrhythmische Medikamente wenig effektiv sind. Das Auftreten von AT ist mit einer erhöhten Morbidität und Mortalität assoziiert [75, 76]. AT stellen bei Fontan-Patientinnen, die ein intrinsisch erhöhtes Thromboserisiko aufweisen, eine Indikation zur Antikoagulation dar [18].

#### Bradyarrhythmien

Sinusknotendysfunktion als Ursache für symptomatische Bradyarrhythmien sind häufiger als AV-Blockierungen (in etwa 2/3 zu 1/3) und treten ebenfalls häufiger bei Personen mit atriopulmonalem Fontan als bei lateralem Tunnel-Fontan und extrakardialem Conduit auf. Aufgrund der Abhängigkeit des Herzzeitvolumens von der Herzfrequenz bei relativ fixiertem Schlagvolumen werden Bradyarrhythmien hämodynamisch oft schlecht toleriert [75, 76, 81]. Häufig findet sich ein ektoper atrialer oder langsamer junktionaler Ersatzrhythmus. Letzterer kann durch Verlust der AV-Synchronität eine zusätzliche hämodynamische Verschlechterung der Fontan-Physiologie bedingen. Bradykardien begünstigen auch das Auftreten von AT [81]. AV-Blockierungen sind vor allem mit der zugrunde liegenden Anatomie des univentrikulären Herzfehlers assoziiert und treten in 30 % perioperativ auf [81].

#### Ventrikuläre Tachykardien

Die wahre Inzidenz von VT bei Personen mit Fontan-Physiologie ist nicht bekannt, und SCD Ereignisse werden häufig auch durch AT und Bradyarrhythmien verursacht [76]. SCD-Ereignisse treten bereits in jungem Alter (median 18. bis 20. Lebensjahr) auf [81]. Unter intensiviertem Rhythmusmonitoring wurden VT und auch nicht anhaltende ventrikuläre Tachykardien (nsVT) häufiger (insgesamt bei 11 % der Patienten) detektiert und waren mit einem erhöhten Risiko für Herztransplantation und Tod assoziiert [82]. Allerdings liegen keine ausreichenden und zuverlässigen Daten vor, die eine Prädiktion von VT und SCD-Ereignissen bei EMAH mit Fontan-Zirkulation ermöglichen [8, 18].

#### Behandlungsoptionen

Die Vorstellung von Personen mit Fontan-Kreislauf in der NFA erfolgt eventuell nicht aufgrund von tachykarden Palpitationen, sondern aufgrund einer zunehmenden Herzinsuffizienz [76, 80]. Die Diagnose von Tachyarrhythmien kann oft schwierig sein, aufschlussreich ist ein Vergleich mit einem Vor-EKG [17, 80]. Persistierende, hämodynamisch instabile Tachykardien – auch hämodynamisch instabile AT – sollten ehestmöglich kardiovertiert werden. Eine elektrische Kardioversion ist üblicherweise schneller und effektiver und einem medikamentösen Kardioversionsversuch vorzuziehen [8, 17, 18]. Bei implantiertem Schrittmachersystem oder ICD kann ein atriales Overdrivepacing versucht werden [8]. Da häufig gleichzeitig eine Neigung zu Bradyarrhythmien vorliegt, sollte die Möglichkeit zur raschen Initiierung einer Interims-Schrittmacherstimulation vorhanden sein. Zu beachten ist hier, dass bei PatientInnen mit einer Fontan-Physiologie der transvenöse Zugang zum Herz limitiert ist. Bei atriopulmonalem Fontan oder lateralem Tunnel-Fontan ist er nur bis in den Vorhof möglich, bei einem Fontan mit extrakardialem Conduit hingegen gar nicht. In letzterem Fall bleibt nur die Option des transkutanen Pacings oder eines retrograden transaortalen Zuganges mit Pacing im Systemventrikel [17]. Beim Platzieren der Klebeelektroden ist auf mögliche atypische anatomische Situationen (Situs inversus, Dextrokardie) zu achten [80]. Im Falle hämodynamisch gut tolerierter AT können eine Frequenzkontrolle und Antikoagulation eingeleitet und eine Überstellung an das betreuende EMAH-Zentrum organisiert werden [17, 76, 80]. Ein Thrombenausschluss mit transösophagealer Echokardiographie vor geplanter Kardioversion ist bei hämodynamisch stabilen Patienten angeraten [9]. Zur langfristigen Kontrolle von AT ist oft ein multifaktorieller Therapieansatz mit Sanierung pathologischer anatomischer Substrate, antiarrhythmischer Medikation, Katheterablation und Schrittmachertherapie erforderlich [80, 83]. Betablocker und Klasse-III-Antiarrhythmika können als Rezidivprophylaxe von Tachyarrhythmien versucht werden, allerdings sind Rezidive unter medikamentöser Therapie häufig. Das Risiko für die Entwicklung einer Hyperthyreose unter Amiodaron ist gerade bei jungen Menschen mit Fontan-Zirkulation hoch, und der potenzielle negativ inotrope Effekt von Sotalol ist im Falle einer eingeschränkten Kontraktilität des systemischen Ventrikels zu berücksichtigen [18, 80, 83]. Frühzeitig sollte eine Katheterablation angestrebt werden, allerdings ist die Erfolgsrate niedrig, und Rezidiveingriffe sind häufig notwendig. Elektrophysiologische Untersuchungen sollten nur an einem Zentrum mit Erfahrung in der Behandlung von EMAH vorgenommen werden. Zur Durchführung ist einerseits die Kenntnis der anatomischen kardialen Situation erforderlich, andererseits müssen technische Möglichkeiten vorhanden sein, mit den Kathetern durch Punktion des Fontan-Baffles oder mittels elektromagnetischer Navigation retrograd über die Aorta in den PV-Vorhof zu gelangen [8, 18, 80, 83]. Da AT häufig durch Bradykardien induziert sind, kann eine atriale Stimulation mit Erhöhung der Vorhoffrequenz eine Reduktion der Arrhythmieepisoden bewirken oder auch eine höhere Dosierung der antiarrhythmischen Medikation ermöglichen [80, 81]. Aufgrund des limitierten transvenösen Zuganges und des hohen Thromboembolierisikos subaortal gelegener Sonden werden im Falle einer erforderlichen permanenten Gerätetherapie standardmäßig epikardiale Systeme chirurgisch implantiert [8, 81]. Neben der Indikation zur Schrittmacherimplantation aufgrund von symptomatischen Bradyarrhythmien (Sinusknotendysfunktion, AV-Blockierungen) werden Schrittmachersysteme in der Hälfte der Fälle zur Kontrolle von AT implantiert [76, 81]. Nicht AV-synchrone und ventrikuläre Stimulation erhöhen langfristig das Risiko für Herzinsuffizienz und Insuffizienz der AV-Klappen. Stimulation des systemischen Ventrikels über eine apikal platzierte epikardiale Sonde oder die primäre Anlage von 2 ventrikulären Sonden zur Synchronisation der ventrikulären Stimulation sollten erwogen werden. Allerdings kann sich die Sondenimplantation aufgrund von Vernarbungen durch Voroperationen technisch schwierig gestalten [75, 76, 81]. Eine eindeutige Empfehlung zur Defibrillatorimplantation besteht nur bei PatientInnen nach überlebtem SCD oder einer anhaltenden VT nach Ausschluss reversibler Ursachen (I C) [8, 18]. Bei PatientInnen mit hochgradig reduzierter Funktion des singulären Ventrikels und weiteren Risikofaktoren (Herzinsuffizienzsymptome entsprechend NYHA Klasse II oder III, nsVT oder höhergradiger AV-Klappeninsuffizienz oder einer QRS-Dauer > 140 ms) kann ebenfalls eine ICD-Implantation erwogen werden (IIb C) [18], allerdings müssen mögliche technische Schwierigkeiten bei der Geräteimplantation, eine hohe Rate an inadäquaten Schockabgaben und die psychische Belastung der PatientInnen unbedingt mitbedacht werden [8, 76]. Neben konventionellen ICD mit epikardial implantierten Sonden kann ein subkutaner ICD von Vorteil sein, da dieser ohne Sternotomie implantiert werden kann. Allerdings ist über diesen aktuell keine antibradykarde Stimulation möglich [76, 81].

### 6. Seltenere Vitien mit spezifischen Charakteristiken

#### a. Ebstein-Anomalie

**Abb. 7:**
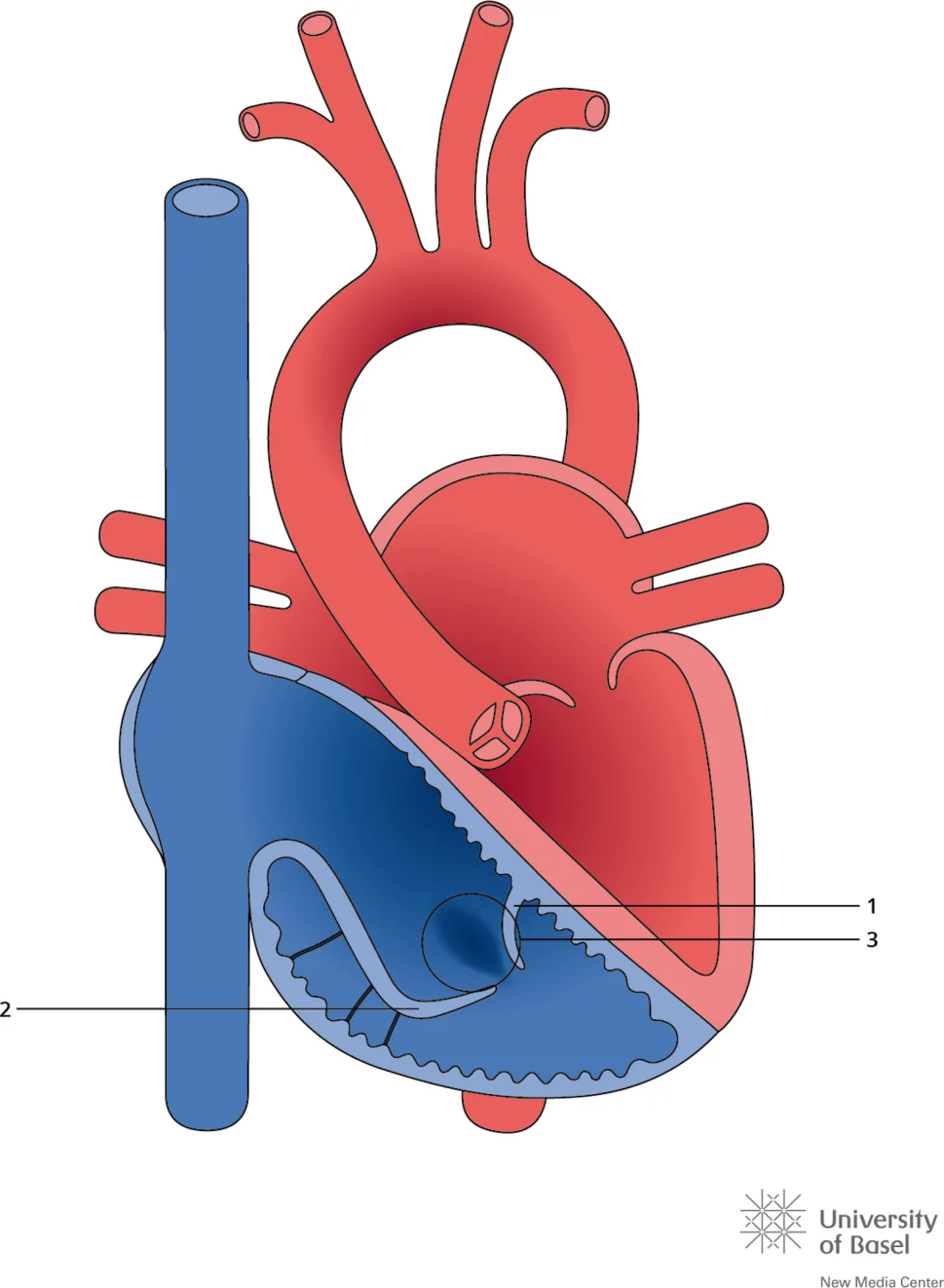
Ebstein-Anomalie [89], *1* apikale Verlagerung des septalen Segels, *2* verlängertes anteriores Segel mit anhaftenden Sehnenfäden, *3* Trikuspidalklappeninsuffizienz

Die Ebstein-Anomalie ist eine seltene Fehlbildung mit inkompletter Delamination der Trikuspidalklappe vom rechtsventrikulären Myokard, welche in einer gering bis stark ausgeprägten apikalen Verlagerung des funktionellen Anulus des septalen und posterioren Segels resultiert, das anteriore Segel ist meist vergrößert (Abb. 7; [18]). Daraus resultieren eine Trikuspidalklappeninsuffizienz unterschiedlichen Schweregrades, eine Region mit „atrialisierten“ dysplastischen Anteilen des rechten Ventrikels und eine teils massive Dilatation des rechten Vorhofs. Meist findet sich auf Vorhofebene entweder ein ASD oder ein persistierendes Foramen ovale. Dadurch kann ein Rechts-Links-Shunt mit resultierender Zyanose vorliegen und ein erhöhtes Risiko für paradoxe Embolien darstellen [18, 84]. Die besseren Ergebnisse der Trikuspidalklappenrekonstruktion mittels Cone-Repair im Vergleich zu früheren Operationsmethoden führten dazu, dass an erfahrenen chirurgischen Zentren bei hochgradiger Trikuspidalklappeninsuffizienz zu einem früheren Zeitpunkt bereits eine Operation durchgeführt wird [18, 84]. Vor einem Verschluss des Vorhofshunts muss eine Evaluierung erfolgen, inwieweit dies eine Drucksteigerung im rechten Vorhof oder eine Verminderung des Herzzeitvolumens bedingen kann [18].

##### Supraventrikuläre Tachykardien

Bis zu 38 % der PatientInnen mit Ebstein-Anomalie haben akzessorische Leitungsbahnen, vor allem entlang des septalen und posterioren Anteils des Trikuspidalklappenrings [7, 12, 84]. In wiederum bis zu 50 % der Fälle finden sich multiple akzessorische Leitungsbahnen, was mit einem erhöhten Risiko für SCD verbunden ist [7, 18]. Auch AV-nodale Reentrytachykardien und atriofaszikuläre Fasern kommen vor. Sekundäre atriale Arrhythmien sind IART, Vorhofflattern und seltener Vorhofflimmern auf Basis einer Dilatation des rechten Vorhofs, welche mit zunehmendem Alter häufiger werden [84, 85]. Bei bereits vorliegender rechtsventrikulärer Dysfunktion können AT hämodynamisch schlecht toleriert werden sowie zu einer Zunahme des Rechts-Links-Shunts und damit der Zyanose führen. Eine rasche Terminierung der Tachykardie sollte daher angestrebt werden. Medikamente, die den AV-Knoten verlangsamen, sollten vermieden werden, wenn akzessorische Leitungsbahnen vorhanden sind [7, 84]. Als Langzeittherapie werden bei symptomatischen AT und auch bei Präexzitation im EKG eine elektrophysiologische Untersuchung und Katheterablation empfohlen, wenngleich die Erfolgsraten niedriger und das Rezidivrisiko höher sind [8, 18]. Ebenso wird vor jeder geplanten operativen Trikuspidalklappenrekonstruktion zur Durchführung einer elektrophysiologischen Abklärung geraten, um eventuell vorhandene Arrhythmiesubstrate (v. a. akzessorische Leitungsbahnen) zu identifizieren und zu abladieren, da diese postoperativ einer interventionellen Therapie möglicherweise nicht mehr zugänglich sind [18, 84].

##### Ventrikuläre Tachyarrhythmien

Monomorphe VT sind seltene Makro-Reentrytachykardien aus dem dysplastischen Myokard des RV, der durch die Verlagerung der Trikuspidalklappenöffnung auf der atrialen Seite liegt. Als Folge einer rechtsventrikulären Dilatation und Dysfunktion können im Langzeitverlauf sekundäre ventrikuläre Arrhythmien, meist polymorphe VT und Kammerflimmern auftreten [84]. Eine spezifische Empfehlung hinsichtlich primärprophylaktischer ICD-Implantation existiert nicht und muss in Einzelfällen individuell diskutiert werden [84].

#### b. Kongenital korrigierte Transposition der großen Arterien (ccTGA)

**Abb. 8:**
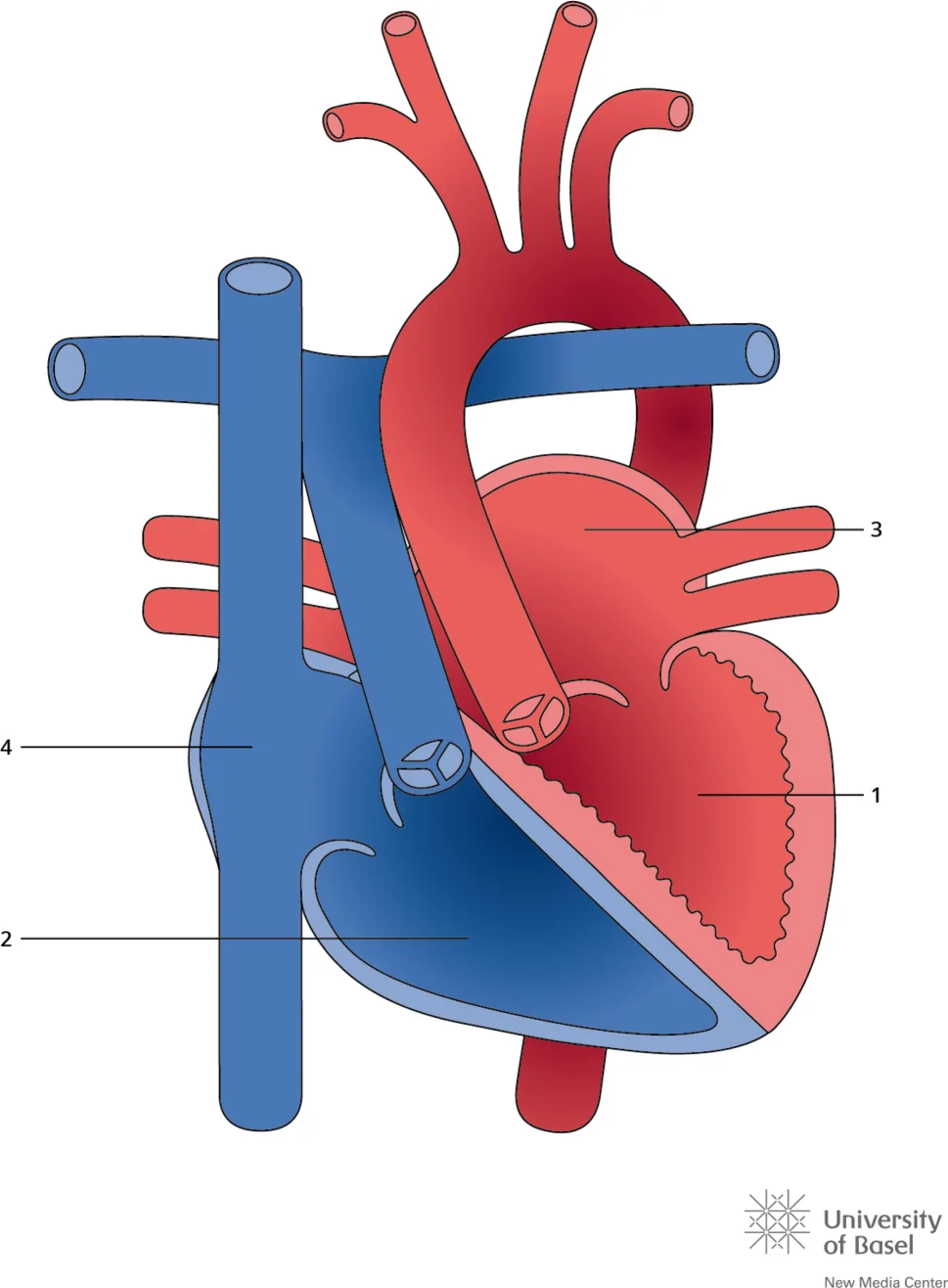
Kongenital korrigierte Transposition der großen Arterien [89], *1* anatomisch rechter Ventrikel, *2* anatomisch linker Ventrikel, *3* linker Vorhof, 4 rechter Vorhof

Die kongenital korrigierte Transposition der großen Arterien ist ein seltener angeborener Herzfehler, bei dem sowohl eine atrioventrikuläre als auch eine ventrikuloarterielle Diskordanz vorliegen. Dies entspricht zwar einer physiologischen Korrektur (kongenital korrigiert), jedoch ist an den anatomisch rechten Vorhof der anatomisch linke Ventrikel angeschlossen, aus dem die Pulmonalarterie entspringt. Umgekehrt liegt der anatomisch rechte Ventrikel in systemischer Position zwischen linkem Vorhof und unterhalb der Aorta (Abb. 8; [86]). Im Falle eines normalen Situs befinden sich der anatomisch linke Ventrikel und die Mitralklappe rechts und der anatomisch rechte Ventrikel und die Trikuspidalklappe links [86]. Die Anatomie der SVC, IVC und Lungenvenen ist üblicherweise normal. Häufig sind jedoch andere Läsionen, wie zum Beispiel ein VSD, eine linksventrikuläre (subpulmonale) Ausflusstraktstenose oder Trikuspidalklappenanomalien assoziiert [86]. Das Reizleitungssystem ist abnormal, und das jährliche Risiko zur Entwicklung eines AV-Blockes liegt bei 2 % [18]. Der AV-Knoten in der üblichen Position an der Spitze des Koch-Dreieckes ist häufig hypoplastisch und nicht mit dem AV-Bündel verbunden. Ein zweiter AV-Knoten liegt anterior im Bereich der Kontinuität von Mitral- und Pulmonalklappe mit Verbindung zu einem penetrierenden His-Bündel, das auf der rechten Seite des Ventrikelseptums rund um den Anulus der Pulmonalklappe verläuft und – wenn vorhanden – am Oberrand eines VSD. Danach teilt sich dieses His-Bündel in invertierte Tawara-Schenkel auf [10]. Das Risiko im Langzeitverlauf eine Herzinsuffizienz zu entwickeln ist aufgrund der subaortalen/systemischen Position des anatomisch rechten Ventrikels hoch. Im Falle der Notwendigkeit einer ventrikulären Stimulation sollte daher eine biventrikuläre Stimulation angestrebt werden. Varianten des Ostiums des Koronarsinus, vor allem das Vorhandensein mehrerer Ostien und das komplette Fehlen des Koronarsinus, sind beschrieben. Häufig mündet der Koronarsinus jedoch an bekannter Position in den rechten Vorhof [86, 87]. Zu beachten ist auch, dass eine Fixierung der ventrikulären Sonde im subpulmonalen anatomisch linken Ventrikel aufgrund der glatten Septumwand oft nur mit einer Schraubelektrode gelingt [18]. Vorhofarrhythmien, vor allem Reentrytachykardien und Arrhythmien aufgrund von akzessorischen Leitungsbahnen, sind mit der spezifischen Anatomie des Reizleitungssystems assoziiert und erfordern bei der Durchführung einer Ablation ein spezifisches Fachwissen sowie ein genaues Mapping des Reizleitungssystems [10, 86]. Bei zunehmender Herzinsuffizienz kommen auch ventrikuläre Arrhythmien vor [9].

#### c. Heterotaxiesyndrome

Heterotaxiesyndrome beschreiben eine komplexe anatomische Situation mit abnormaler Lateralisation der thorakoabdominellen Organe entlang der Links-Rechts-Achse, wobei paarige Strukturen symmetrisch gespiegelt angelegt sind [88]. In Bezug auf die kardiale Situation bedeutet das, dass entweder 2 Vorhöfe mit anatomischen Charakteristiken eines rechten Vorhofes (rechter Isomerismus) oder linken Vorhofes (linker Isomerismus) vorhanden sind, meist mit assoziierten Anomalien der Mündungen von systemischen (SVC und IVC) und Lungenvenen [88]. In Bezug auf die AV-Verbindung bestehen häufig komplexe Vitien mit biventrikulärer (z. B. AVSD oder rotierte Ventrikel, d. h. anatomisch linker Ventrikel rechtsseitig und anatomisch rechter Ventrikel linksseitig) oder univentrikulärer Morphologie [88].

##### Rechter Isomerismus

Fast immer sind bilaterale Sinusknoten in beiden rechten Vorhöfen vorhanden. Ebenso sind der AV-Knoten und die Reizleitungsbahnen doppelt angelegt („twin AV node“), welche distal im Ventrikel fusionieren können („conduction sling“). Es besteht eine Prädisposition zu AV-nodalen Reentrytachykardien [10].

##### Linker Isomerismus

Der Sinusknoten (üblicherweise eine rechtsseitige Struktur) kann fehlen, dysplastisch oder ektop gelegen sein. Auch besteht die Neigung zur AV-Blockierung [10].

Unbedingt muss die Situation der venoatrialen Verbindungen vor einer elektrophysiologischen Untersuchung oder einer Schrittmacherimplantation abgeklärt werden. Häufig ist bei beiden Varianten eine doppelt angelegte obere Hohlvene. Typisch für den rechten Isomerismus sind Fehlmündungen der Lungenvenen und ein Fehlen des Koronarsinus, während die IVC normal angelegt ist. Im Falle eines linken Isomerismus liegt häufig eine unterbrochene IVC vor, die untere Körperhälfte drainiert über eine posterior im Thorax laufende V. azygos oder hemiazygos in die SVC [88].

#### d. Vitien mit persistierenden Shunts und zyanotische Vitien

Die hämodynamische Signifikanz von Shuntläsionen hängt von mehreren Faktoren ab, unter anderem von der Größe des Defektes und den Druckverhältnissen in den durch den Defekt verbundenen Herzkammern. Üblicherweise besteht bei einfachen Shuntvitien wie ASD und restriktiven VSD ein Links-Rechts-Shunt. Bei komplexeren Vitien mit Vorliegen einer pulmonalarteriellen Hypertonie (Eisenmenger-Syndrom) oder einer Pulmonalstenose kann es zu einer Shuntumkehr und damit zu einem Rechts-Links-Shunt und Zyanose kommen [18]. Eine Hypotension wie z. B. im Rahmen von Tachyarrhythmien kann die Zyanose durch Verstärkung des Rechts-Links-Shunts verschlechtern [7]. Da das Risiko einer paradoxen Embolie besteht, muss beim Verabreichen von Medikamenten über venöse Zugänge darauf geachtet werden, keine Luftbläschen einzubringen. Hierfür gibt es spezielle Luftfilter für venöse Verweilkanülen oder Zentralvenenkatheter. Intrakardiale Sonden bei Gerätetherapien erhöhen das Risiko für systemische Thromboembolien. Eine adäquate Thromboseprophylaxe wird in diesen Fällen empfohlen. Bei Indikation zur permanenten Gerätetherapie sollten eher epikardiale Systeme implantiert werden, wenn Korrektureingriffe bzw. Shuntverschlüsse nicht möglich oder kontraindiziert sind. Ansonsten richtet sich die Therapie von Tachy- und Bradyarrhythmien nach der zugrunde liegenden Anatomie und ist im entsprechenden Kapitel beschrieben [7–9, 18].

## Data Availability

Alle dieser Arbeit zugrunde liegenden Daten sind in diesem Artikel enthalten.

## Abkürzungen

AHF: Angeborener Herzfehler
ASD: Atrialer Septumdefekt
AT: Atriale Tachykardie
AV: Atrioventrikulär
AVB: Atrioventrikuläre Blockierung
AVSD: Atrioventrikulärer Septumdefekt
CcTGA: Kongenital korrigierte Transposition der großen Arterien
CRT: Kardiale Resynchronisationstherapie
CT: Computertomographie
d‑TGA: (Komplette) Transposition der großen Arterien
eCV: Elektrische Kardioversion
EKG: Elektrokardiogramm
EMAH: Erwachsene mit angeborenem Herzfehler
IART: Intraatriale Reentrytachykardien
ICD: Implantierbarer Kardioverter-Defibrillator
IVC: Vena cava inferior
LVOT: Linksventrikulärer Ausflusstrakt
MRT: Magnetresonanztomographie
NFA: Notfallambulanz
NOAK: Neue orale Antikoagulanzien
nsVT: Nicht anhaltende ventrikuläre Tachykardie
OAK: Orale Antikoagulation
PSM: Implantierter Schrittmacher
PV: Pulmonalvenen
RVOT: Rechtsventrikulärer Ausflusstrakt
SCD: Plötzlicher Herztod (sudden cardiac death)
SVC: Vena cava superior
SVT: Supraventrikuläre Tachykardie
TCPC: Totale cavopulmonale Anastomose
TOF: Fallot-Tetralogie
VSD: Ventrikelseptumdefekt
VT: Ventrikuläre Tachykardie
WCD: Wearable-Kardioverter-Defibrillator
3D: Dreidimensional
